# Supplementary material for: Uncertainty-aware machine learning to predict non-cancer human toxicity for the global chemicals market
Source: Nat Commun. 2026 Jan 7;17:647. doi: 10.1038/s41467-025-67374-4 (PMC12816579; doi:10.1038/s41467-025-67374-4)
Supplement: Supplementary file 1 — Supplementary Information [file 41467_2025_67374_MOESM1_ESM.pdf]

Supplemental information 1

Uncertainty-aware machine learning to predict non-cancer human toxicity for the global chemical market

Kerstin von Borries[1], Katie V Beckwith[2], Jonathan M Goodman[2], Weihsueh A Chiu[3], Olivier Jolliet[1], and Peter Fantke[1,4,5,6*]*

[1] Quantitative Sustainability Assessment, Department of Environmental and Resource Engineering, Technical University of Denmark, Bygningstorvet 115, 2800 Kgs. Lyngby, Denmark

[2] Centre for Molecular Informatics, Yusuf Hamied Department of Chemistry, University of Cambridge, Lensfield Road, Cambridge CB2 1EW, United Kingdom

[3] Department of Veterinary Physiology and Pharmacology, Texas A&M University, 4466 TAMU, College Station, TX 77843-4466, USA

[4] substitute ApS, Graaspurvevej 55, 2400 Copenhagen, Denmark

[5] Department for Evolutionary Ecology and Environmental Toxicology, Goethe University, 60438 Frankfurt am Main, Germany

[6] Department of Environmental Sciences, College of Agriculture and Environmental Sciences, University of South Africa, Florida 1710, Roodepoort, South Africa

Contents

1. Data preprocessing 2
   1.1. Data and SMILES curation 2
   1.2. Features 3
2. Conventional ML model training without uncertainty quantification 4
   2.1. Conventional ML algorithms 4
   2.2. Cross-validation procedure 5
   2.3. Consensus model building 6
   2.4. Conventional ML performance results 7
3. Uncertainty-aware ML model training 11
   3.1. Uncertainty frameworks 11
   3.2. Cross-validation procedure 12
   3.3. Comparison with conventional ML models 13
   3.4. UAM performance and calibration results 14
4. Model application 33

References 36

1. Data preprocessing

1.1. Data and SMILES curation

We trained our models using the large dataset of probabilistic PODs derived by Aurisano et al. (2023) through a statistical approach based on curated in vivo effect data from the US EPA Toxicity Value Database (ToxValDB). The dataset contained PODs for reproductive/developmental (rd) and general non-cancer (nc) effects via chronic oral exposure for more than 10,000 chemicals that act as surrogates for PODs derived through authoritative assessment. The separate endpoints allow considering the differing severity between lifelong reproductive/developmental impairments and general non-cancer effects that typically manifest later in life, when used, for example, in comparative impact assessment[1]. We complemented the dataset with 26 general non-cancer PODs for dioxin-like chemicals that were not yet part of the dataset by leveraging toxic equivalency factors (TEFs) reported by DeVito et al. (2024)[2], thereby improving the representation of chemicals with low general non-cancer PODs (signifying high toxicity). For every chemical in the dataset, chemical structure information in the form of SMILES was collected from the US EPA Comptox Chemicals Dashboard[3] and complemented with results from the open chemistry database PubChem[4] using the python package PubChemPy[5] and the PubChem Identifier exchange service.[6] Structures were identified primarily via their CAS registration numbers and secondarily via their chemical names or abbreviations. Following harmonization and the related evident duplicate removal, CAS numbers with multiple valid SMILES (n=133) were inspected to select the most appropriate structure based on information provided in the data set. In the case of ambiguous or missing information, the simplest / non-stereometric form was chosen. Inversely, multiple CAS with the same associated SMILES were inspected selecting the most representative record for the given structure. This affected 150 structures with reported POD_{rd} and 175 structures with reported POD_{nc}. This approach yielded unique CAS-SMILES combinations covering 4,832 chemicals in the reproductive/developmental dataset and 5,370 chemicals in the general non-cancer dataset. Subsequently, both datasets were filtered to keep PODs that were obtained from a minimum of 4 underlying measured data points to reduce data-related uncertainty as Aurisano et al. derived surrogate PODs using the 25th percentile. The resulting data sets consisted of 3,402 chemicals with POD_{rd} and 2,717 chemicals with POD_{nc} (expanded dataset). After standardizing chemical structures using the protocol by Mansouri et al. (2018), which includes the removal of inorganic and organometallic compounds, large molecules (>1000 g/mol), and the stripping of salts and solvents, 2,357 unique chemicals with POD_{rd} and 1,945 with POD_{nc} were available for model training and validation (standardized dataset). In addition, the expanded data set was used to assess the potential for achieving robust uncertainty quantification for a more diverse chemical space than applicable for conventional ML models including non-standardized chemicals. The subset of non-standardized chemicals was also used as a challenging external test set for the models trained on standardized chemicals to assess how uncertainty quantification behaves in entirely unfamiliar chemical domains. All PODs were converted from mg/kg-d to log10-transformed units of mol/kg-d prior to training representing the rational link between molecules and their biological activity,[7] though we note that such conversion have been shown to have little influence in other modeling studies.[8] Prediction results were presented in mg/kg-d for easier interpretation.

Supplementary Figure **1** shows a histogram of the expanded datasets for POD_{rd} and POD_{nc}, highlighting the three most toxic compounds.

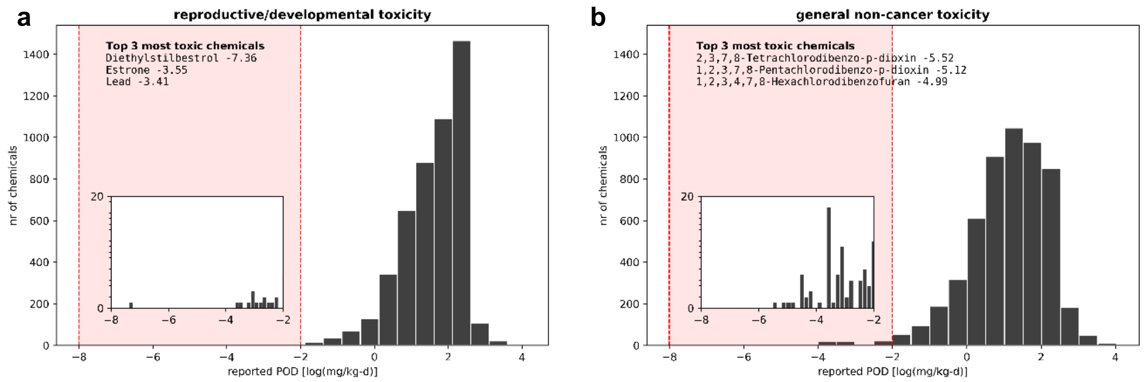

Supplementary Figure 1. Histogram of (a) reported reproductive/developmental (rd) and (b) general non-cancer (nc) PODs highlighting toxic outliers with POD < 0.01 mg/kg-d representing 0.4% and 1.5% of training chemicals with reported POD_{rd} and POD_{nc}, respectively. Source data are provided as a Source Data file.

1.2. Features

We considered four types of molecular descriptors that numerically describe chemical structures to be used as training features for the ML models. We chose two types of fingerprints (MACCS, Morgan) and two types of continuous descriptors (RDKit, CDDD) (see Supplementary Table 1 for more details). The RDKit descriptors as well as the MACCS and Morgan fingerprints were calculated with RDKit v2022.9.5.[9] Morgan fingerprints were calculated with a length of 512 bits and a radius of 2, following initial tests that showed that increasing fingerprint length (up to 4,096) and radius (up to 4) did not help improve modelling performance. The CDDD embedding was calculated with the CDDD python package provided by Winter et al. (2019).[10]

While MACCS and RDKit descriptors are knowledge-based descriptors that encode structural fragments or properties of chemical structures, Morgan fingerprints and CDDD embedding provide an unweighted representation of the full chemical structure. We removed highly correlated features (Pearson correlation coefficient > 0.9) from the knowledge-based descriptors but not from the structural representations as these provide a valid representation only when used together. In addition, we applied a Yeo-Johnson transformation to the numeric RDKit descriptors to achieve Gaussian-like distributions and scale all RDKit descriptors to zero mean and unit variance. To avoid data leakage in cross-validation, all feature processing steps are learned from training sets and applied accordingly to the respective test sets within each cross-validation fold (see sections S1.2b and S1.3b for details).

Supplementary Table 1. Brief overview of the four types of molecular descriptors used in this study including the number of features for each type and their primary references.

**Type**	**Number**	**Description**	**Reference**
MACCS fingerprints	166	binary, structural keys of pre-defined sub-structures and fragments	Durant et al. (2002)[11]
Morgan fingerprints	adjustable	binary, circular extended-connectivity fingerprint (ECFP) covering all possible structural fragments in each atom neighborhood (up to a defined radius), hashed to a defined length (bits)	Rogers & Hahn (2010)[12]
RDKit descriptors	208	continuous/distinct 1D and 2D descriptors covering fragment counts, topological, structural, and physicochemical parameters	RDKit (2022)[9]
CDDD embedding	512	Continuous auto-encoded representation of chemical structure obtained from the latent space of a SMILES translation model based on a recurrent neural network (RNN)	Winter et al. (2019)[10]

2. Conventional ML model training without uncertainty quantification

We performed a systematic evaluation of conventional ML algorithms without uncertainty quantification to benchmark performance of best-practice ML model development protocols training each algorithm with the four types of molecular descriptors

2.1. Conventional ML algorithms

We selected six algorithms that cover common algorithm families following different functional principles for finding an optimal fit to a given training data set: Multiple linear regression (MLR), k nearest neighbor (KNN), support vector machine (SVM), random forest (RF), extreme gradient boosting machine (XGB) and a simple feed forward neural network (NN) (see Supplementary Table 2 for details). MLR, KNN, SVM and RF were implemented using scikit-learn v1.2.2. The SVM was trained with a radial basis kernel function (RBF) for highly flexible, non-linear behavior. The XGB was implemented using xgboost v2.0.0. The NN was implemented with Tensorflow v2.13.0 with one hidden layer with the same number of neurons as the input layer, following initial tests that showed no significant performance improvements with wider (up to twice the number of input features) and deeper (up to 5 hidden layers) architectures (see Supplementary Figure 5). The hidden layer was activated with a rectified linear unit (ReLU) to enable non-linear behavior. The network was trained for 500 epochs with the Adam optimizer using an optimizable learning rate and a learning rate scheduler that reduced the learning rate by a factor of 0.5 if the validation loss did not reduce by at least $1e^{-5}$ mean squared error (MSE) for 10 epochs (patience). In addition, early stopping was implemented if the validation loss did not reduce by at least $1e^{-5}$ MSE in 20 epochs restoring the weights from the best previous epoch. Learning rate scheduler and early stopping callbacks operated based on a 20% validation split. For regularization, we optimized drop-out at the input layer and weight decay during the nested cross-validation (see Supplementary Table 3 for details). The Tensorflow model was made compatible with scikit-learn functionalities using wrappers by scikeras v0.11.0.

Supplementary Table 2. Brief overview of six types of machine learning algorithms used in this study including their functional principle and their primary references.

Family	Algorithm	Abbr.	Principle	References
Multiple linear regression	Elastic net	MLR	Ordinary least squares regression with a tuneable mix of L1 (Lasso) and L2 (Ridge) regularization	Zou & Hastie (2004)
Instance-based learning	K nearest neighbor regression	KNN	memory-based, non-parametric algorithm that predicts a new data point based on the (weighted) average of the most similar instances (nearest neighbors) in the training data set	Cover & Hart (1967)[13]
Support vector machine	Support vector regression	SVM	Vapnik-Chervonenkis (VC) theory-based algorithm that fits to the training data considering an error-insensitive tube around the fitted function with different kernel options for non-linear behaviour	Boser et al. (1992)[14]
Ensemble (bagged)	Random forest	RF	ensemble of parallel decision trees trained on bootstrapped data samples that predicts a new data point by aggregating predictions from underlying decision trees	Breiman (2001)[15]
Ensemble (boosted)	Extreme Gradient Boosting	XGB	ensemble of sequential decision trees each trained on the residual errors of its predecessor thereby tweaking weak	Chen & Guestrin (2016)[16]

			predictions of preceding decision trees (stagewise addition model)	
Neural network	Feed forward neural network	NN	(simple) artificial neural network, consisting of an input layer, one or more hidden layers and an output layer with optional non-linear activations	Rosenblatt (1958)[17]

2.2. Cross-validation procedure

For every descriptor-algorithm combination, model performance was assessed through a nested cross-validation procedure with five inner folds to optimize relevant hyperparameters of the respective algorithm and ten outer folds to robustly estimate the generalization error of the model. Hyperparameters and appropriate value ranges were selected focusing on settings controlling the learning behavior and the complexity of each algorithm (see Supplementary Table 3 for details). The space of hyperparameter combinations was searched using Bayesian optimization with testing (10 × number of hyperparameters) samples in each fold. Bayesian optimization was implemented with scikit-optimize v0.9.0. The best-performing model across the five inner loops was retrained on all available training data in the inner loop and its external prediction performance tested in the outer loop. Chemicals were assigned to each fold using target value-based stratified sampling based on order of magnitude of PODs, where the upper and lower 1%-ile of the POD value range were aggregated into one stratification class each to ensure a sufficient number of samples per class. This approach ensured a similar distribution of POD values across all folds and compensated impacts from data imbalance. The nested cross-validation was repeated ten times to assess the variation in the generalization error across different training data samples (10 outer folds) with different random initial states (10 seeds). The generalization error was assessed using mean absolute error (MAE), median absolute error (MdAE), root mean squared error (RMSE) and the coefficient of determination (R2) as error metrics (see Supplementary Table 4 for detailed definitions). Model performances are compared to a mean predictor model as baseline.

Supplementary Table 3. Overview of hyperparameter optimization including a brief description and the optimization ranges for each algorithm. Parameters that were varied within a logarithmic uniform distribution are denoted with (log).

Algorithm	Hyperparameters	Description
MLR	alpha: 0.0...1.0<br>l1_ratio: 0.0...1.0	regularization strength<br>mix between L1 and L2 regularization
KNN	n_neighbors: 3…20<br>weights: uniform, distance<br>metric: minkowski, jaccard	nr of nearest neighbors<br>uniform or distance-weighted average<br>metric for distance calculation
SVM	gamma: 1e-3...10 (log)<br>C: 0.1...10 (log)<br>epsilon: 0.0...1.0	RBF kernel coefficient<br>regularization strength<br>width of no penalty tube
RF	n_estimators: 100…1000<br>max_depth: 2…20<br>min_samples_leaf: 1…100<br>max_samples: 0.5…1.0<br>max_features: 0.5…1.0<br>min_impurity_decrease: 1e-3...0.1 (log)	number of trees in the forest<br>maximum depth of tree<br>minimum samples required in each leaf node<br>number of samples to train base estimators<br>number of features to consider at each split<br>minimum loss reduction required to split
XGB	n_estimators: 100…1000<br>learning_rate: 0.03...0.3 (log)<br>max_depth: 2...20<br>min_child_weight: 0...10<br>subsample: 0.5...1.0	number of boosting rounds<br>step size shrinkage for weight update<br>maximum depth of tree<br>minimum sum of instance weight in child<br>training sample ratio per boosting iteration

	colsample_bytree: 0.5...1.0<br>gamma: 1e-3...0.1 (log)	column sample ratio for creating each tree<br>minimum loss reduction required to split
NN	batch_size: 32, 64, 128<br>dropout_rate: 0.0…0.5<br>learning_rate: 1e-4…0.01 (log)<br>weight_decay: 1e-5…0.1 (log)	number of samples per weight update<br>ratio of randomly ignored input nodes<br>step size shrinkage for weight update<br>ratio of weight shrinkage per iteration

Supplementary Table 4. Overview of error metrics used to assess generalization performance of different machine learning methods.

Metric	Abbr.	Formula	Definitions
Mean absolute error	MAE	$\frac{1}{n}\sum_{i=1}^{n}\lvert y_i - \hat{y}_i\rvert$	n: number of data points<br>y_i: measured value for i^{th} data point<br>$\hat{y}_i$: predicted value for i^{th} data point<br>$\bar{y}$: mean measured value across data points
Median absolute error	MdAE	median $\lvert y_i - \hat{y}_i\rvert$	
Root mean squared error	RMSE	$\sqrt{\frac{1}{n}\sum_{i=1}^{n}(y_i - \hat{y}_i)^2}$	
Coefficient of determination	R2	$1 - \frac{\sum_{i=1}^{n}(y_i - \hat{y}_i)^2}{\sum_{i=1}^{n}(y_i - \bar{y})^2}$	

2.3. Consensus model building

To establish a single, high-performing benchmark for the uncertainty-aware models, we created a consensus model by taking the average across the subset of conventional ML models with the highest statistically significant prediction performances ($p < 0.05$). To estimate the generalization errors and test the statistical significance of the differences in model performance we followed Nadeau and Bengio (2000) to perform pairwise correlated two-sided student's t-tests. The procedure for a single pairwise correlated t-test between models A and B following K-fold cross-validation with J repetitions is briefly outlined below:

1. Compute the prediction error (loss) for models A and B for each data point i and repetition j (we used the squared loss).

   $z_{i,j}^{A} = \left(\hat{y}_{i,j}^{A} - y_{i,j}\right)^2 \quad | \quad z_{i,j}^{B} = \left(\hat{y}_{i,j}^{B} - y_{i,j}\right)^2$

2. Estimate the mean difference $\hat{d}$ in generalization error from the mean loss difference between models A and B across all J repetitions.

   $d_j = \frac{1}{n_j}\sum_{i=1}^{n_j} z_{i,j}^{A} - z_{i,j}^{B}$

   $\hat{d} = \frac{1}{J}\sum_{j=1}^{J} d_j$

3. Estimate the variance in the difference in the generalization error between models A and B accounting for the correlation induced due to overlapping training sets among the crossvalidation folds.

   $\hat{\sigma}^2 = \frac{1}{J-1}\sum_{j=1}^{J}\left(d_j - \hat{d}\right)^2$

$$\tilde{\sigma}^2 = \left(\frac{1}{J} + \frac{\rho}{1-\rho}\right) \cdot \hat{\sigma}^2 \quad \text{where } \rho = \frac{1}{K}$$

4. Compute the p-value for the null hypothesis that models A and B perform identically from the cumulative student's t-distribution function.

$$p = 2 \cdot \text{cdf}(-|\hat{t}| \mid \nu = J - 1, \mu = 0, \sigma = 1) \quad \text{with } \hat{t} = \frac{\hat{d}}{\tilde{\sigma}}$$

Consensus model predictions were obtained by averaging across all models with high prediction performance that were not statistically distinguishable from the model with the lowest mean RMSE ($p<0.05$). Based on the results from the statistical tests, the models comprising the consensus models are given in Supplementary Table 5.

Supplementary Table 5. Overview of conventional ML models building the consensus model when trained on the standardized data set for reproductive/developmental and general non-cancer PODs.

**POD endpoint**	**Models building consensus model** (algorithm-descriptor combination)
Reproductive/developmental toxicity	SVM-CDDD<br>XGB-RDKIT
General non-cancer toxicity	RF-MACCS<br>SVM-CDDD<br>SVM-MACCS<br>KNN-MACCS<br>XGB-MACCS<br>XGB-RDKIT

2.4. Conventional ML performance results

This section presents results comparing the prediction performance of the conventional ML algorithms in combination with different molecular descriptors. Supplementary Figure 2 and Supplementary Figure 3 compare the mean prediction performance and its variance across repeated cross-validation folds for the selected metrics for POD_{rd} and POD_{nc}, respectively. The best performance was generally achieved with knowledge-based descriptors (RDKit, MACCS), while SVM also achieved competitive performance using the embedding-based CDDD. Supplementary Figure 4 shows the prediction performance of the obtained consensus model predictions. The highly flexible algorithms SVM, XGB and RF generally outperformed KNN, MLR and our simple NN. Supplementary Figure 5 shows that NN performance did not substantially improve when increasing architecture complexity in terms of number and size of hidden layers. All models outperform the mean predictor model (represented by the dotted line) across all metrics, except for RMSE in the POD_{rd} predictions, where all models exhibit RMSE values exceeding the baseline on some folds. Another exception is the NN model trained with RDKit descriptors to predict POD_{rd} which showed negative R2 values on some folds.

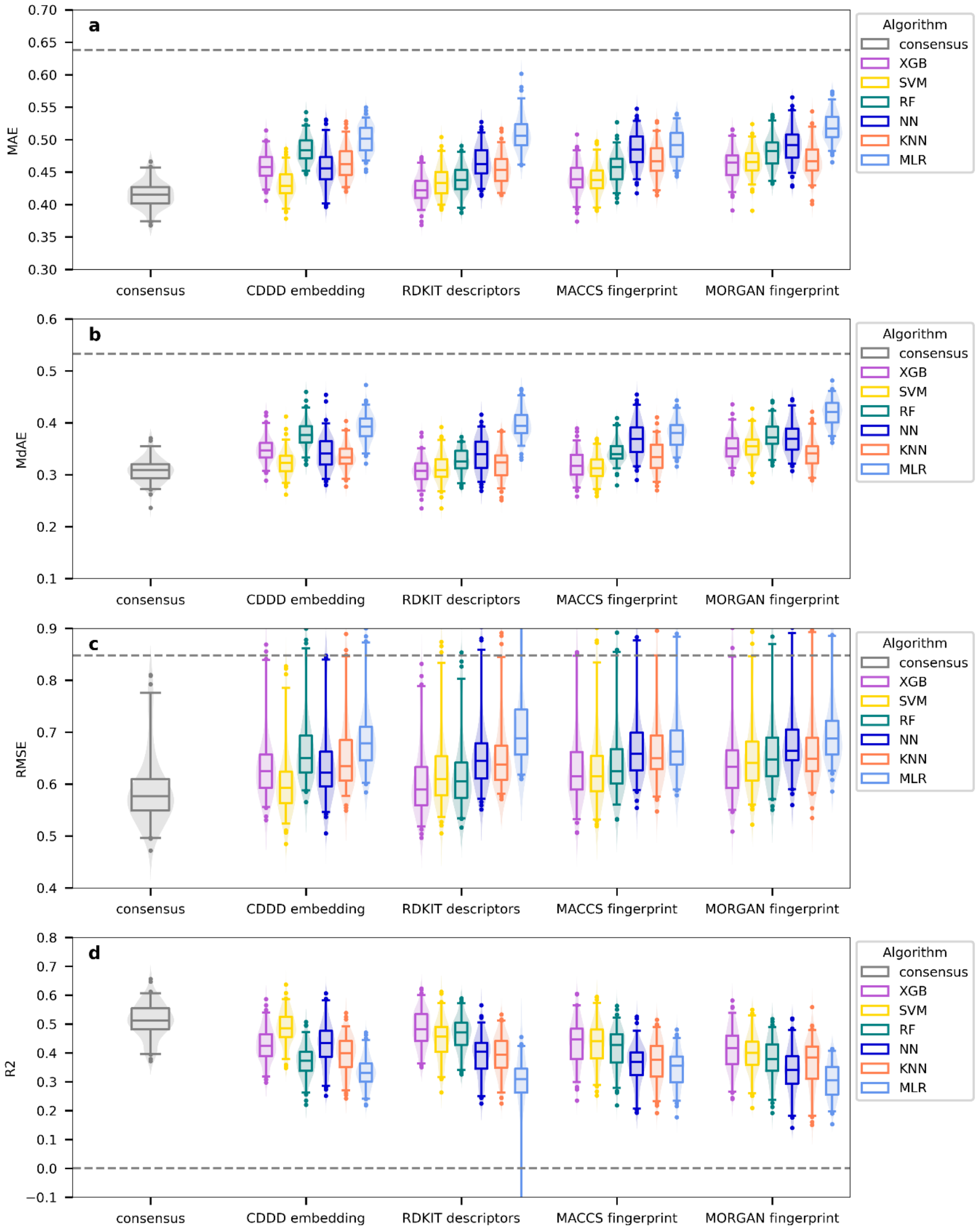

Supplementary Figure 2. Performance comparison of conventional ML models and the consensus model (see color legend) for predicting reproductive/developmental PODs across crossvalidation folds for four metrics (a) mean absolute error (MAE), (b) median absolute error (MdAE), (c) root mean squared error (RMSE), and (d) coefficient of determination (R2). The dotted line represents the performance of the mean predictor model. A lower MAE, MdAE and RMSE indicates higher performance, while a higher R2 indicates higher performance. Boxplots show the median (center) and interquartile range (boxes) with whiskers extending to the 2.5th and 97.5th percentiles. Source data are provided as a Source Data file.

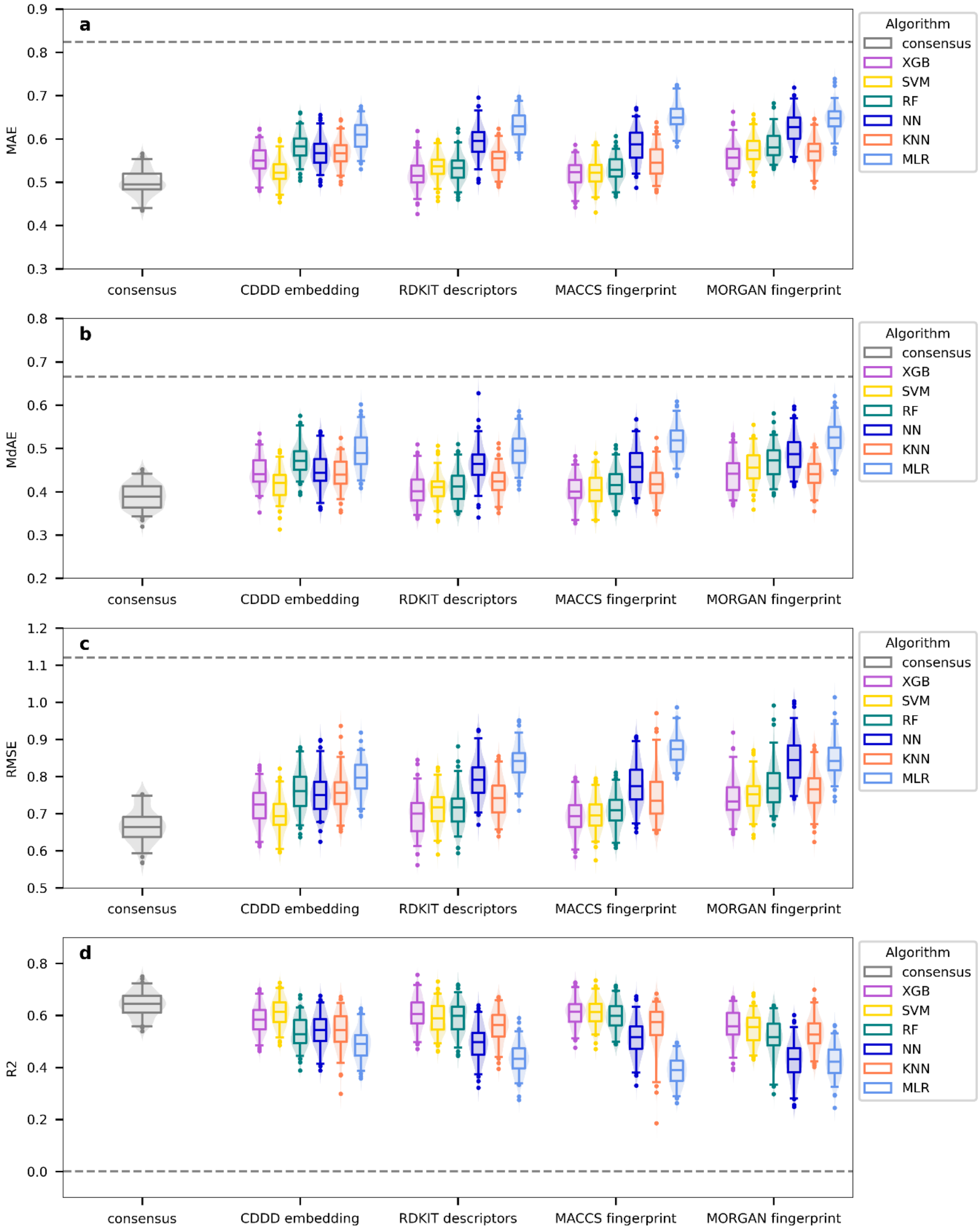

Supplementary Figure 3. Performance comparison of conventional ML models and the consensus model (see color legend) for predicting general non-cancer PODs across crossvalidation folds for four metrics (a) mean absolute error (MAE), (b) median absolute error (MdAE), (c) root mean squared error (RMSE), and (d) coefficient of determination (R2). The dotted line represents the performance of the mean predictor model. A lower MAE, MdAE and RMSE indicates higher performance, while a higher R2 indicates higher performance. Boxplots show the median (center) and interquartile range (boxes) with whiskers extending to the 2.5th and 97.5th percentiles. Source data are provided as a Source Data file.

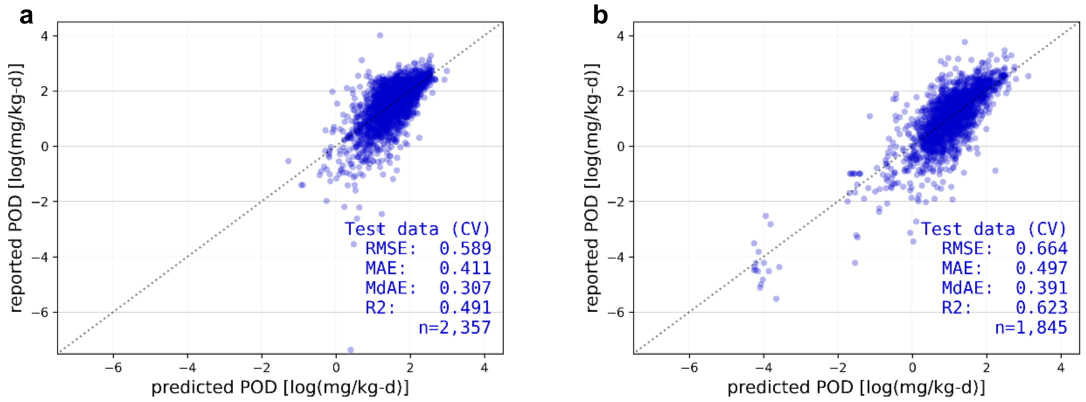

Supplementary Figure 4. Prediction performance of the consensus models for (a) reproductive developmental and (b) general non-cancer PODs. Source data are provided as a Source Data file.

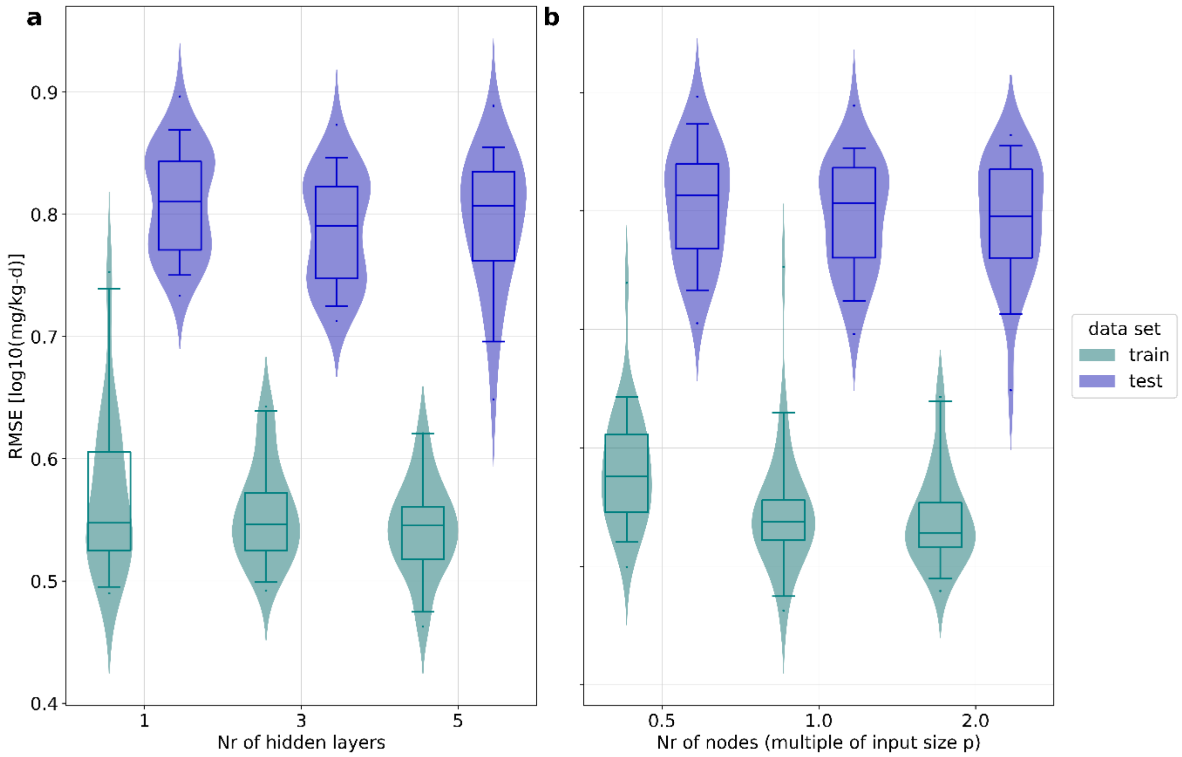

Supplementary Figure 5. Changes in POD_{nc} prediction error (given by RMSE) for different neural network architectures in terms of (a) number of hidden layers and (b) size of hidden layers. Hidden layer sizes are defined relative to the input size (*p*) as multiples of *p*. The figure shows that changes towards deeper and wider architecture did not lead to substantial reductions in prediction error on training set (green) or cross-validated test set (blue). Boxplots show the median (center) and interquartile range (boxes) with whiskers extending to the 2.5th and 97.5th percentiles. Source data are provided as a Source Data file.

3. Uncertainty-aware ML model training

3.1. Uncertainty frameworks

Common approaches to convey model uncertainty often cover only one type of uncertainty. A commonly reported metric describing prediction uncertainty is the average prediction error. When obtained through cross-validation, the prediction error provides a robust estimate of a model's generalization error on unseen data. This metric is mainly concerned with aleatoric uncertainty. In addition, as a summary metric it does not inform about potential variability in uncertainty across predictions, called heteroscedasticity. Some machine learning methods allow capturing data-related heteroscedasticity, e.g. quantile regression-based approaches, providing prediction intervals based on empirically estimated quantiles. Other machine learning methods provide an uncertainty estimate with each point prediction capturing epistemic uncertainty, for example ensemble-based approaches that provide the variance across point predictions produced by a large set of predictors. Taken alone, these uncertainty estimates tend to be overconfident as they fail to adequately account for either epistemic or aleatoric uncertainty. In this study we compared two distinct paradigms within machine learning for quantifying uncertainty, conformal prediction (CP) and Bayesian neural networks (BNNs).

Conformal prediction

CP is rooted in hypothesis testing following classical, frequentist statistics.[18] It builds confidence intervals around point estimates based on a desired confidence level thereby controlling the fraction of erroneous predictions with a statistically valid probability. CP offers a model-agnostic and non-parametric approach that can be combined with any algorithm and provides confidence intervals without assuming a specific uncertainty distribution. However, not all CP-based methods provide heteroscedastic uncertainty quantification. In conformal regression, various conformity score definitions are available, each with different coverage guarantees and computational costs. Most commonly used conformity scores do not adapt well to heteroscedastic data, resulting in constant or minimally varying confidence intervals across prediction points. In response, Romano et al. (2019) introduced conformalized quantile regression (CQR), which effectively adapts to varying degrees of aleatoric uncertainty.[19] However, CQR falls short in distinguishing between aleatoric and epistemic uncertainty leading (among other things) to unreliable coverage of out-of-distribution data. Addressing this, Rossellini et al. (2023) proposed uncertainty-aware conformalized quantile regression (UACQR), refining CQR to effectively address aleatoric and epistemic uncertainty.[20] They included two alternative approaches to estimating epistemic uncertainty – a scaling-based approaches that scales the upper and lower bounds by an explicit estimate of the quantile's standard deviations (UACQR-S) and a percentile-based approach where updated upper and lower bounds are obtained as percentiles from an ensemble of quantile estimates (UACQR-P). In this study, we present results obtained with UACQR-S due to better performance on the calibration metrics. Rossellini et al. implemented their method with four core algorithms: a linear quantile regression, a light gradient boosting machine with quantile loss, a quantile regression forest (qRF) and a quantile regression neural network with two hidden layers. In this study we used qRF as the model algorithm based on our conventional ML model comparison that showed RF among the high-performing algorithms and due to its more comprehensive implementation. We implemented UACQR using the python code provided by Rossellini et al. with a small modification to include median predictions in the model outputs alongside lower and upper bounds. The UACQR model was trained with a 95% confidence level, providing the 2.5th and 97.5th percentiles alongside the median as model outputs. By fitting the inverse cumulative distribution function of a skewed normal distribution to these three percent points, we could express the prediction uncertainty as a standard deviation when necessary.

Bayesian neural network

BNNs apply Bayesian principles to model uncertainty and are the main representative for probabilistic modelling approaches.[18] BNNs move beyond deterministic outputs by attaching probability distributions to each model parameter (biases and weights) and returning a probability distribution for the output prediction itself. This allows them to effectively capture both aleatoric and epistemic uncertainty. We trained our BNN with variational inference implemented in TensorFlow Probability (TFP) using Bayes-by-backprop and negative log-likelihood loss.[21] We defined our BNN with one hidden layer with ReLU activation and an output layer with two nodes assigned to the mean and standard deviation of a Gaussian distribution. A softplus function with scaling factor is used for the standard deviation to ensure positivity with computational stability. Following Blundell et al. (2015)[22], the weights and biases of the hidden and output layers were defined by a variational posterior distribution that approximates the true posterior with a multivariate normal distribution. The prior distribution of the weights and biases were defined as the mixture of two zero-mean normal distributions with respectively small and large variances that allow concentrating parameters close to zero without restricting learning flexibility. Our BNN was trained with the Adam optimizer with a learning rate of 0.002 for 1,000 epochs with callbacks for early stopping and learning rate scheduling as defined for the base NN. Probabilistic model parameters are inherently regularized, rendering additional regularization methods such as drop-out and weight decay unnecessary in BNNs.[22] Prediction results were assessed based on random samples of 500 draws from the predicted output distributions.

3.2. Cross-validation procedure

We set up a cross-validation procedure to robustly estimate the prediction performance and uncertainty calibration for each of the two uncertainty modelling approaches. The cross-validation consisted of ten folds with the random states initialized with a seed of 42. Chemicals were assigned to each fold using target value-based stratified sampling to compensate impacts from data imbalance. Due to their unique structures UACQR and TFP models were not compatible with conventional hyperparameter optimization tools. Based on experience with equivalent conventional ML models, we assigned the qRF in UACQR a maximum depth of 15, considering maximum half of all features and requiring a minimum impurity decrease of 0.001 at each split, thus preventing excessive overfitting. The BNN models were assigned a batch size of 32 and a learning rate of 0.002. The lack of hyperparameter optimization is expected to affect BNNs more than RF-based CP models, as RFs consistently perform well without extensive tuning. NNs, however, are highly sensitive to hyperparameters, as shown in conventional ML optimization, where their settings varied significantly across folds and features. Despite this, the performance trend between CP and BNN UAMs aligned with that of conventional RF and NN models. While using hyperparameters based on experience with conventional ML models trained with different subsets of the same data poses a slight risk of data leakage, the impact is likely minimal: RFs are less affected by hyperparameter choices, and BNNs are more likely to lose performance due to insufficient tuning rather than gain from unintended information. The overall prediction performance was assessed using mean absolute error (MAE), median absolute error (MdAE), root mean squared error (RMSE) and the coefficient of determination (R2) (see Supplementary Table **4** for details).

The quality of the uncertainty quantification was assessed considering three aspects of calibration: confidence-based, error-based, and distance-based calibration. We defined confidence- and error-based calibration curves following Yang & Li (2023)[23]. The confidence-based calibration curve compares the expected confidence level and the observed fraction of measured data points falling within the predicted confidence interval. It can be summarized by the expected calibration error (ECE) which describes the mean absolute deviation between expected and observed fractions (see Supplementary Table **6**). For the CP models, the confidence level is a parameter defined for the construction of the model and thus required training a new model for every confidence level. To limit computational cost, we obtained the confidence-based calibration points of the CP models

based on a single 20% test set. For the BNN models, credible intervals (the Bayesian equivalent to frequentist confidence intervals) were obtained for defined probabilities (equivalent to confidence levels) from the corresponding percentiles of the cross-validated output distribution samples. The error-based calibration curve compares the consistency between the mean observed prediction error and the mean predicted uncertainty across batches of increasing prediction uncertainty. This requires expressing the predicted uncertainty as a standard deviation. For BNN the standard deviation was obtained directly from the model output, while for CP it was obtained by fitting the inverse cumulative distribution function of a skewed normal distribution to the median and CI bounds. To form the batches, the n testing data points are sorted by the predicted uncertainty and divided into B batches containing $\frac{n}{B}$ data points each. The number of batches was set to the entropy-based optimal number of batches obtained as $\sqrt{n}$ [24]. The error-based calibration can be summarized by the expected normalized calibration error (ENCE) defined by Levi et al (2022)[25] which describes the mean absolute deviation between observed error and predicted uncertainty across batches (see Supplementary Table 6). As ENCE has been shown to be sensitive towards the chosen number of batches [24] we assessed the change in ENCE as a function of number of batches ranging from 5 to 200 (see Supplementary Figure 6). Inspired by the out-of-distribution analysis by Yin et al (2023)[26], we defined a distance-based calibration curve to investigate the uncertainty calibration for chemicals that are structurally different from training chemicals. It assesses the consistency between mean structural dissimilarity and mean prediction uncertainty across batches of increasing prediction uncertainty, applying the same binning approach as for error-based calibration. The structural dissimilarity was described by the average Jaccard distance of each test chemical from its five nearest neighbors among training chemicals based on Morgan fingerprints (1,024 bits, radius 2). The strength of correlation in all calibration curves was indicated by Pearson and Spearman correlation coefficients (see Supplementary Table 6 for details).

Supplementary Table 6. Overview of metrics used to assess calibration quality of the uncertainty modelling approaches.

Metric	Abbr.	Formula	Definitions
Expected calibration error	ECE	$\frac{1}{A}\sum_{a=1}^{A}\lvert \mathrm{CL_a} - f_a \rvert$	A: number of confidence levels<br>$\mathrm{CL_a}$: a^{th} confidence level<br>f_a: fraction of measured points falling into the $\mathrm{CL_a}$ confidence interval
Expected normalized calibration error	ENCE	$\frac{1}{B}\sum_{b=1}^{B}\frac{\lvert \mathrm{RMSE_b} - \mathrm{RMU_b} \rvert}{\mathrm{RMU_b}}$<br>with $\mathrm{RMU_b} = \sqrt{\frac{1}{N_b}\sum_{n_b=1}^{N_b}\sigma_{n_b}^2}$	B: number of batches<br>$\mathrm{RMSE_i}$: RMSE in bin b<br>$\mathrm{RMU_i}$: RMU in bin b<br>N_b: number of data points in bin b<br>σ_{nb}: predicted uncertainty as standard deviation on data point n in batch b
Pearson correlation coefficient	Pearson	$\frac{\mathrm{cov}(\mathbf{X},\mathbf{Y})}{\sigma_X\sigma_Y}$	cov: covariance of two vectors<br>σ_x: standard deviation of vector **X**<br>σ_y: standard deviation of vector **Y**
Spearman correlation coefficient	Spearman	$\frac{\mathrm{cov}(\mathbf{R}[\mathbf{X}],\mathbf{R}[\mathbf{Y}])}{\sigma_{R[X]}\sigma_{R[Y]}}$	**R[·]**: rank variable of ·<br>cov: covariance of two vectors<br>$\sigma_{R[\cdot]}$: standard deviation of rank variable of ·

3.3. Comparison with conventional ML models

To compare the performance of the UAMs to the conventional ML models, we estimated the generalization errors with confidence intervals and assessed the statistical significance of the

differences in model performance using pairwise correlated two-sided student's t-tests. The procedure for a single pairwise correlated t-test between models A and B following *K*-fold cross-validation on a given data set of size *n* without repetition is briefly outlined below:

To determine the confidence interval of the generalization error for a given model:

1. Compute the prediction error (loss) for a given model for each of *n* data points *i* (we used the squared loss).

   $z_i = (\hat{y}_i - y_i)^2$

2. Estimate the generalization error $\hat{z}$ and its variance $\tilde{\sigma}^2$.

   $\hat{z} = \frac{1}{n}\sum z_i \quad | \quad \tilde{\sigma}^2 = \frac{1}{n(n-1)}\sum_{i=1}^{n}(z_i - \hat{z})^2$

3. Determine the $1 - \alpha$ confidence interval of the generalization error from the cumulative student's t-distribution function.

   $z_L = \mathrm{cdf}^{-1}\left(\frac{\alpha}{2}\middle| \nu = n - 1, \hat{z}, \tilde{\sigma}\right) \quad | \quad z_u = \mathrm{cdf}^{-1}\left(1 - \frac{\alpha}{2}\middle| \nu = n - 1, \hat{z}, \tilde{\sigma}\right)$

To assess the difference in performance between two models A and B and the statistical significance:

1. Compute the difference in prediction error (loss) between models A and B.

   $d_i = z_i^A - z_i^B$

2. Estimate the mean difference $\hat{d}$ and its variance $\tilde{\sigma}^2$.

   $\hat{d} = \frac{1}{n}\sum d_i \quad | \quad \tilde{\sigma}^2 = \frac{1}{n(n-1)}\sum_{i=1}^{n}\left(d_i - \hat{d}\right)^2$

3. Compute the t-statistic and its 95% confidence interval for the null hypothesis that models A and B perform identically with $n - 1$ degrees of freedom.

   $\hat{t} = \frac{\hat{d}}{\tilde{\sigma}}$

   $\hat{t}_{2.5\%} = \mathrm{cdf}^{-1}\left(0.025 \mid \nu = n - 1, \mu = \hat{d}, \sigma = \tilde{\sigma}\right)$

   $\hat{t}_{97.5\%} = \mathrm{cdf}^{-1}\left(0.975 \mid \nu = n - 1, \mu = \hat{d}, \sigma = \tilde{\sigma}\right)$

4. Compute the p-value from the cumulative student's t-distribution function.

   $p = 2 \cdot \mathrm{cdf}\left(-\left|\hat{d}\right| \mid \nu = n - 1, \mu = 0, \sigma = \tilde{\sigma}\right)$

5. Compute the effect size using on Cohen's d.

$$d = \frac{\overline{z^A} - \overline{z^B}}{\sqrt{\frac{\left(\tilde{\sigma}^{A^2} + \tilde{\sigma}^{B^2}\right)}{2}}}$$

3.4. UAM performance and calibration results

Supplementary Figure **6** shows the trend in ENCE as a function of the selected number of batches for the CP models trained with RDKit descriptors.

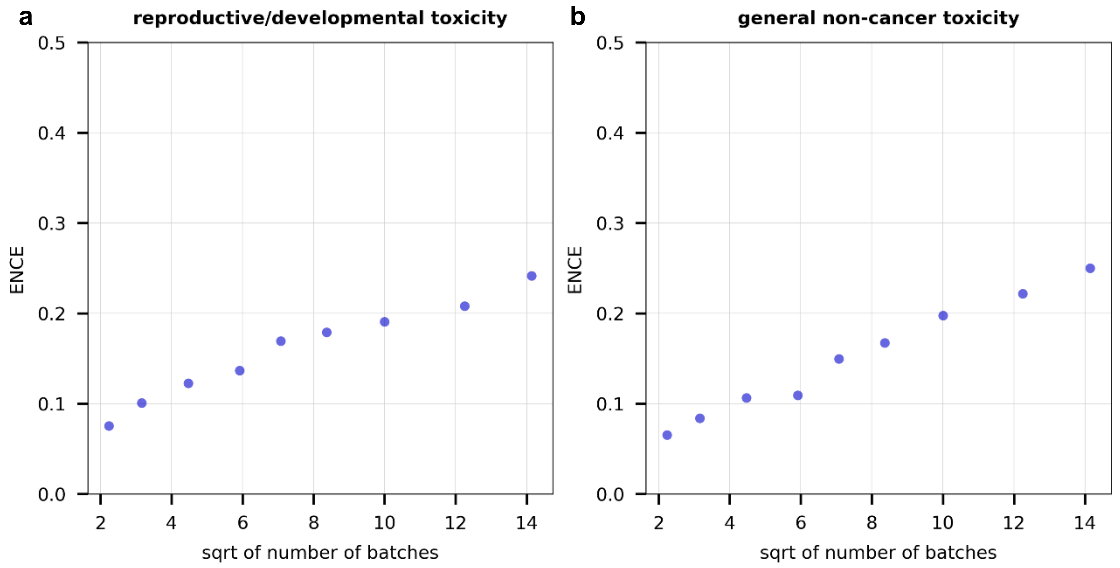

Supplementary Figure 6. ENCE as a function of the square root number of batches for the CP models trained with RDKit descriptors for (a) reproductive/developmental and (b) general non-cancer PODs. Source data are provided as a Source Data file.

Supplementary Figure 7 shows the prediction performance and uncertainty calibration results for the BNN models trained with RDKit descriptors. While both modeling approaches proved well-calibrated for uncertainty quantification along the confidence-based, error-based and distance-based calibration curves, the BNN models had slightly lower prediction performance and provided more conservative estimates of uncertainty compared to CP models.

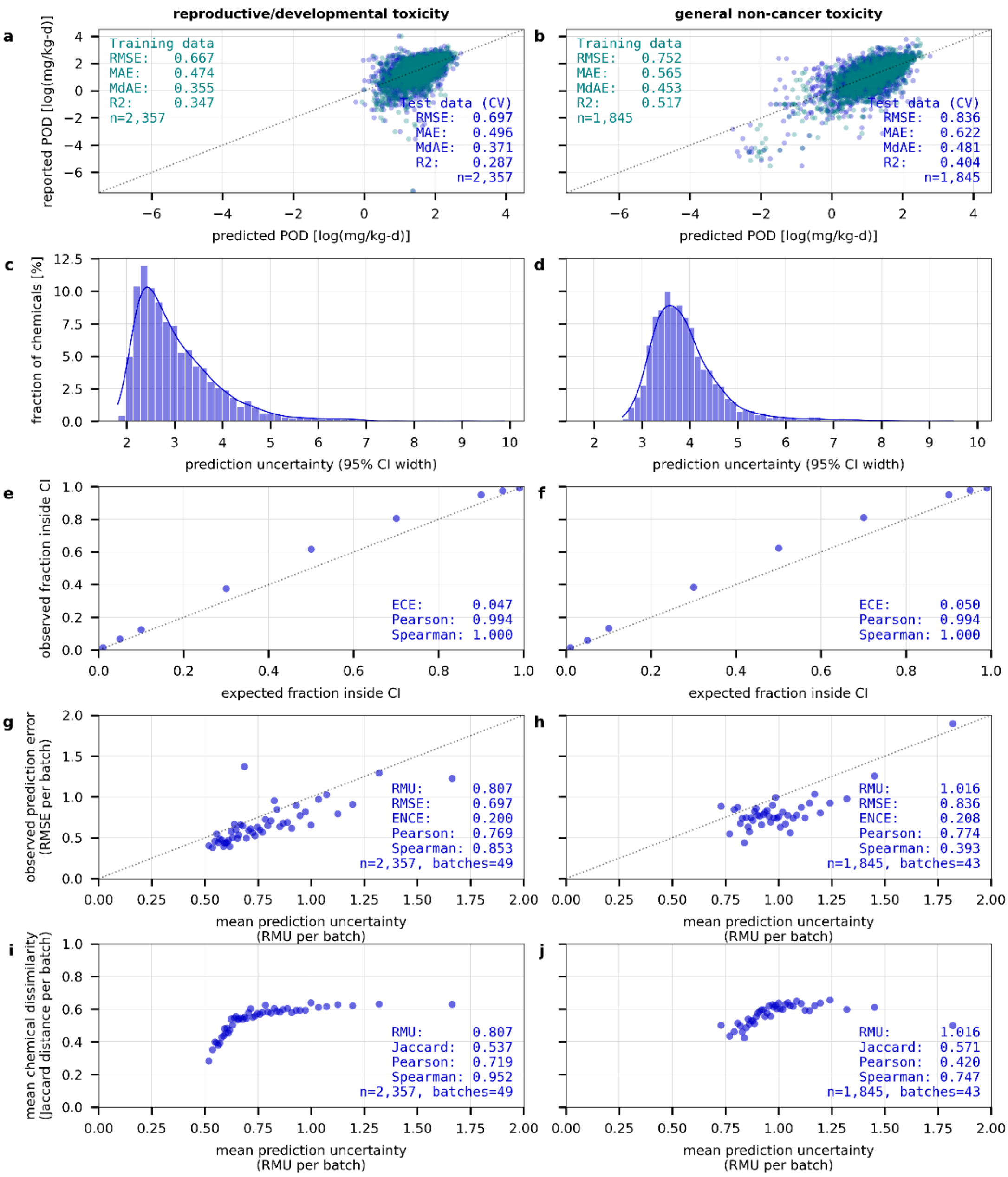

Supplementary Figure 7. Prediction performance and uncertainty calibration of the BNN models trained with RDKit descriptors. **(a, b)** Prediction performance on training data set (green) and cross-validated test data set (blue). **(c, d)** Distribution of prediction uncertainty (95% CI width) **(e, f)** Confidence-based calibration curve **(g, h)** Error-based calibration curve **(I, j)** Distance-based calibration curve. **Abbreviations:** ECE=expected calibration error, ENCE=expected normalized calibration error, CI=confidence interval, CV = cross-validation, Jaccard=mean Jaccard distances from 5 nearest neighbors, MAE=mean absolute error, MdAE=median absolute error, *n*=number of chemicals, Pearson=Pearson correlation coefficient, POD=point of departure, RMSE=root mean squared error, RMU=root mean uncertainty, R2=coefficient of determination, Spearman=Spearman correlation coefficient. Source data are provided as a Source Data file.

Supplementary Figure 8 to Supplementary Figure 13 show the prediction performance and uncertainty calibration results for the CP and BNN models trained with MACCS keys, Morgan fingerprints and CDDD embeddings.

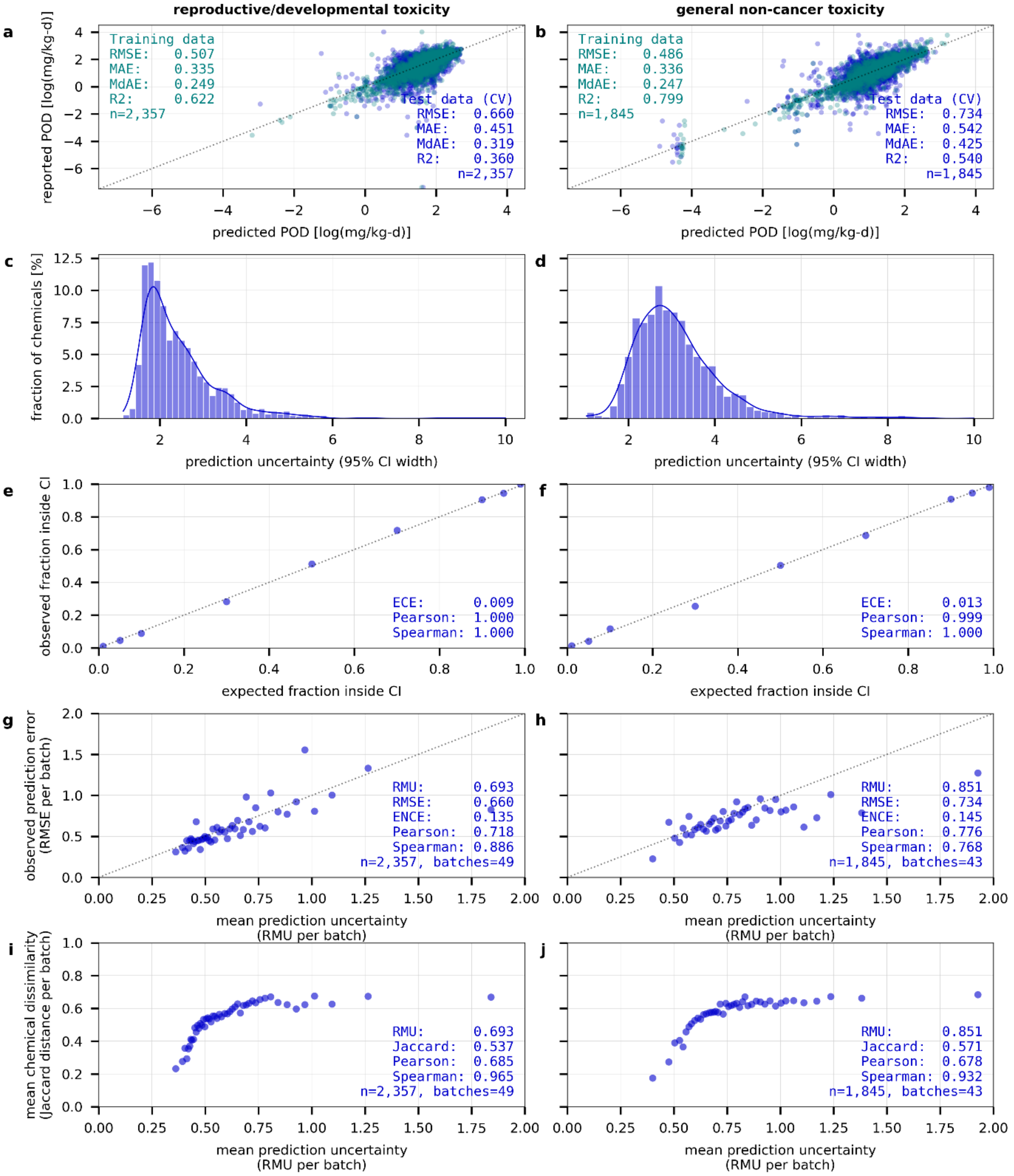

Supplementary Figure 8. Prediction performance and uncertainty calibration of the CP models trained with MACCS keys. **(a, b)** Prediction performance on training data set (green) and cross-validated test data set (blue). **(c, d)** Distribution of prediction uncertainty (95% CI width) **(e, f)** Confidence-based calibration curve **(g, h)** Error-based calibration curve **(i, j)** Distance-based calibration curve. **Abbreviations:** ECE=expected calibration error, ENCE=expected normalized calibration error, CI=confidence interval, CV = cross-validation, Jaccard=mean Jaccard distances from 5 nearest neighbors, MAE=mean absolute error, MdAE=median absolute error, *n*=number of chemicals, Pearson=Pearson correlation coefficient, POD=point of departure, RMSE=root mean squared error, RMU=root mean uncertainty, R2=coefficient of determination, Spearman=Spearman correlation coefficient. Source data are provided as a Source Data file.

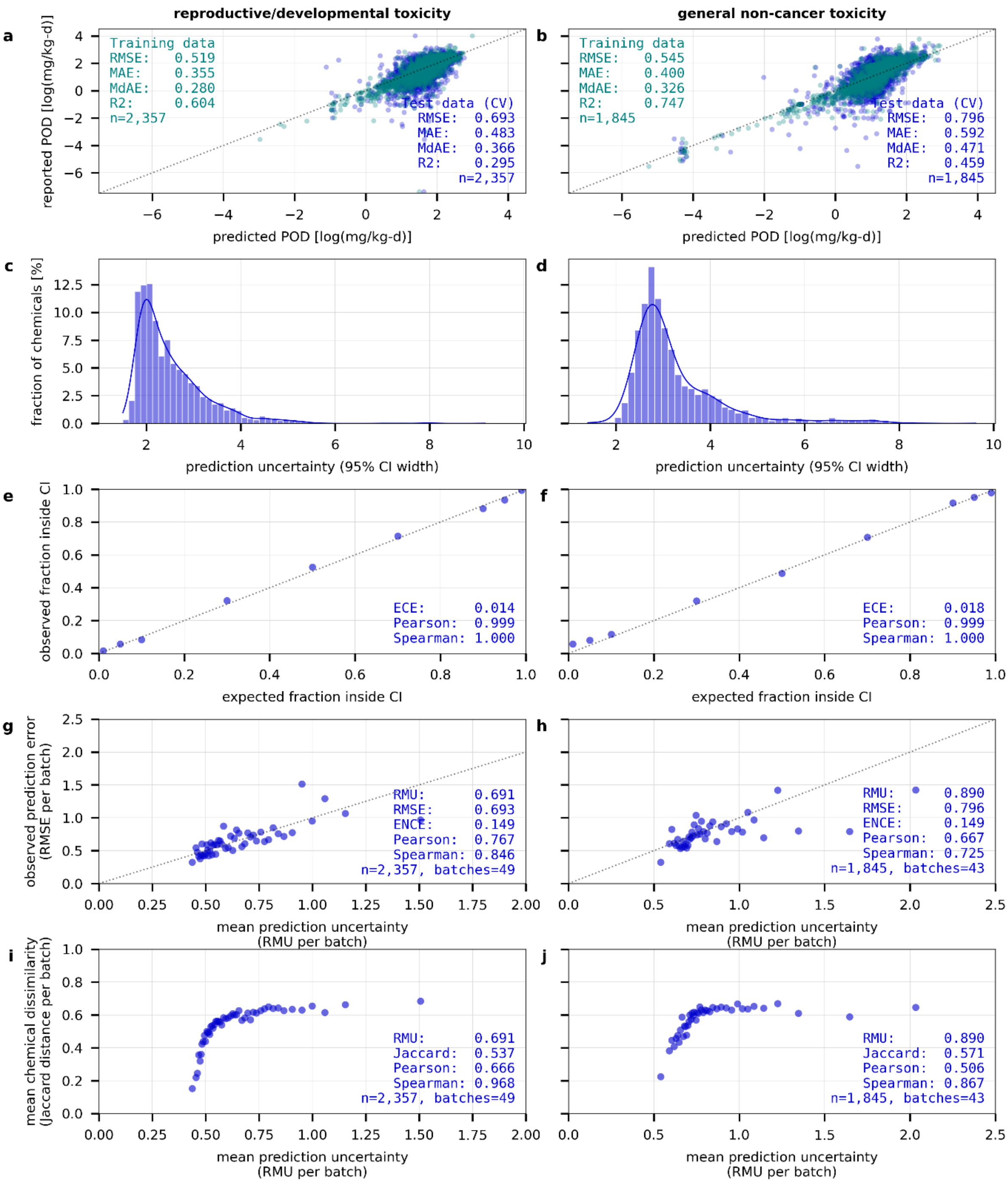

Supplementary Figure 9. Prediction performance and uncertainty calibration of the CP models trained with Morgan fingerprints. **(a, b)** Prediction performance on training data set (green) and cross-validated test data set (blue). **(c, d)** Distribution of prediction uncertainty (95% CI width) **(e, f)** Confidence-based calibration curve **(g, h)** Error-based calibration curve **(i, j)** Distance-based calibration curve. **Abbreviations:** ECE=expected calibration error, ENCE=expected normalized calibration error, CI=confidence interval, CV = cross-validation, Jaccard=mean Jaccard distances from 5 nearest neighbors, MAE=mean absolute error, MdAE=median absolute error, *n*=number of chemicals, Pearson=Pearson correlation coefficient, POD=point of departure, RMSE=root mean squared error, RMU=root mean uncertainty, R2=coefficient of determination, Spearman=Spearman correlation coefficient. Source data are provided as a Source Data file.

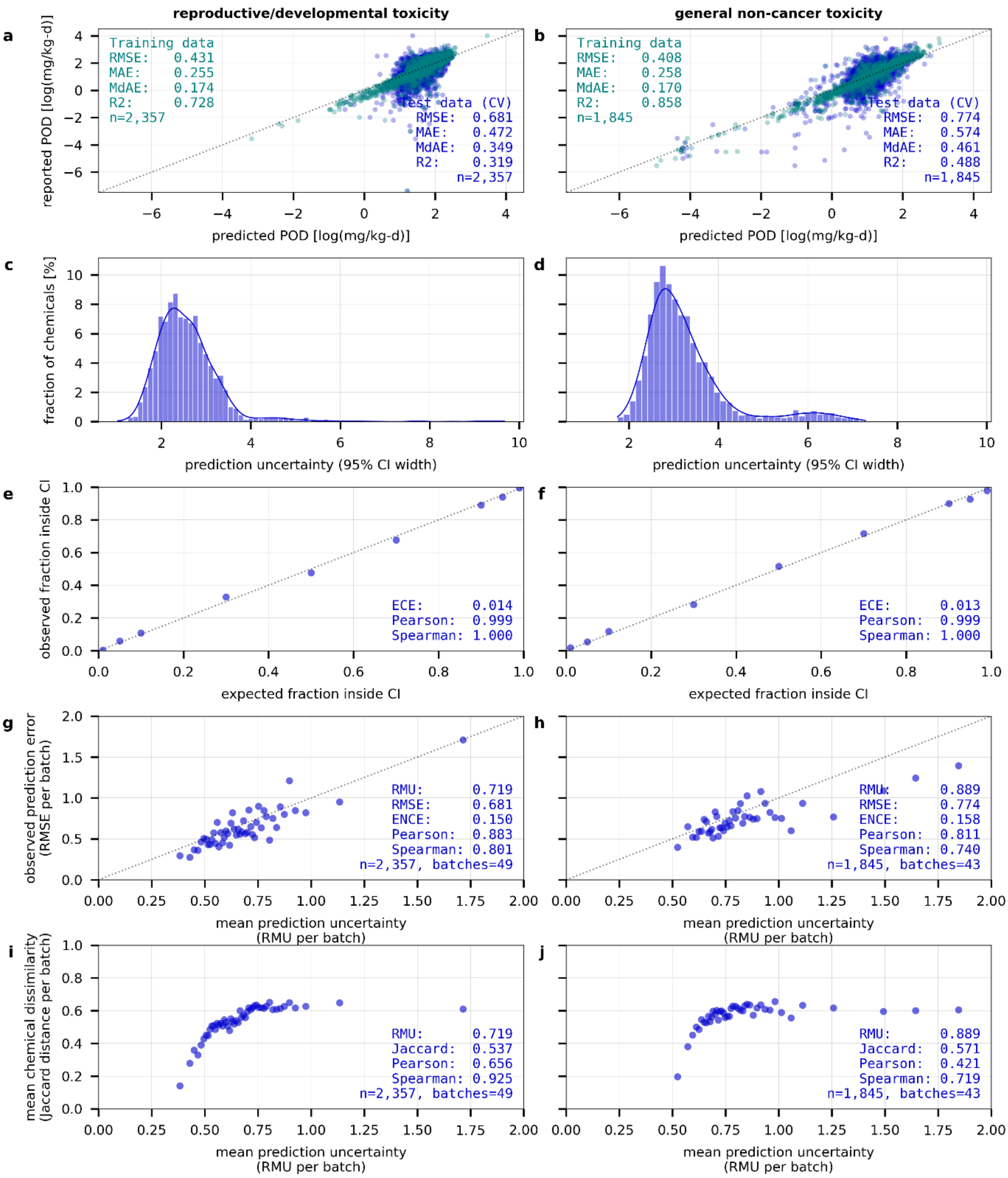

Supplementary Figure 10. Prediction performance and uncertainty calibration of the CP models trained with CDDD embeddings. **(a, b)** Prediction performance on training data set (green) and cross-validated test data set (blue). **(c, d)** Distribution of prediction uncertainty (95% CI width) **(e, f)** Confidence-based calibration curve **(g, h)** Error-based calibration curve **(i, j)** Distance-based calibration curve. **Abbreviations:** ECE=expected calibration error, ENCE=expected normalized calibration error, CI=confidence interval, CV = cross-validation, Jaccard=mean Jaccard distances from 5 nearest neighbors, MAE=mean absolute error, MdAE=median absolute error, *n*=number of chemicals, Pearson=Pearson correlation coefficient, POD=point of departure, RMSE=root mean squared error, RMU=root mean uncertainty, R2=coefficient of determination, Spearman=Spearman correlation coefficient. Source data are provided as a Source Data file.

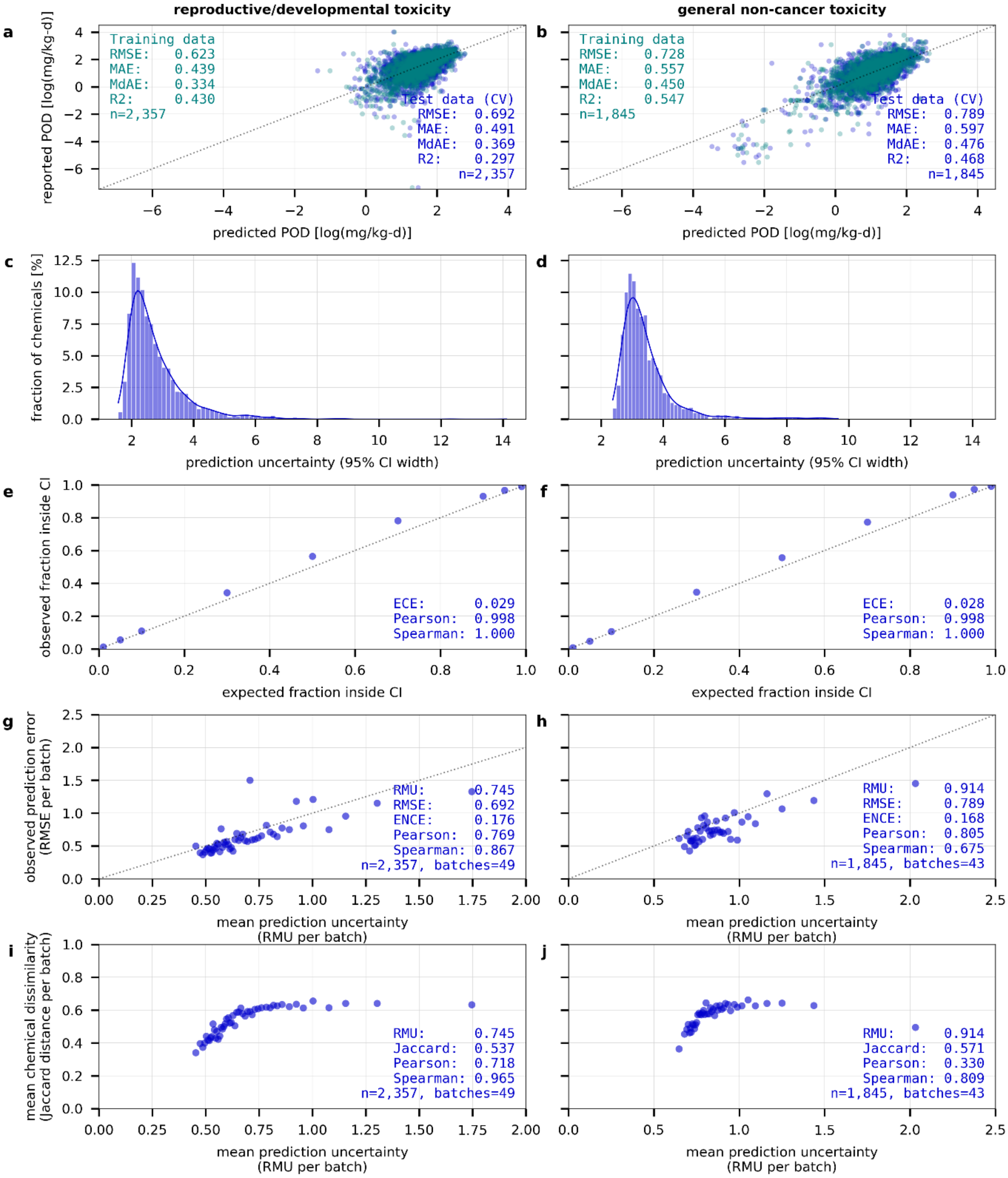

Supplementary Figure 11. Prediction performance and uncertainty calibration of the BNN models trained with MACCS keys. **(a, b)** Prediction performance on training data set (green) and cross-validated test data set (blue). **(c, d)** Distribution of prediction uncertainty (95% CI width) **(e, f)** Confidence-based calibration curve **(g, h)** Error-based calibration curve **(i, j)** Distance-based calibration curve. **Abbreviations:** ECE=expected calibration error, ENCE=expected normalized calibration error, CI=confidence interval, CV = cross-validation, Jaccard=mean Jaccard distances from 5 nearest neighbors, MAE=mean absolute error, MdAE=median absolute error, *n*=number of chemicals, Pearson=Pearson correlation coefficient, POD=point of departure, RMSE=root mean squared error, RMU=root mean uncertainty, R2=coefficient of determination, Spearman=Spearman correlation coefficient. Source data are provided as a Source Data file.

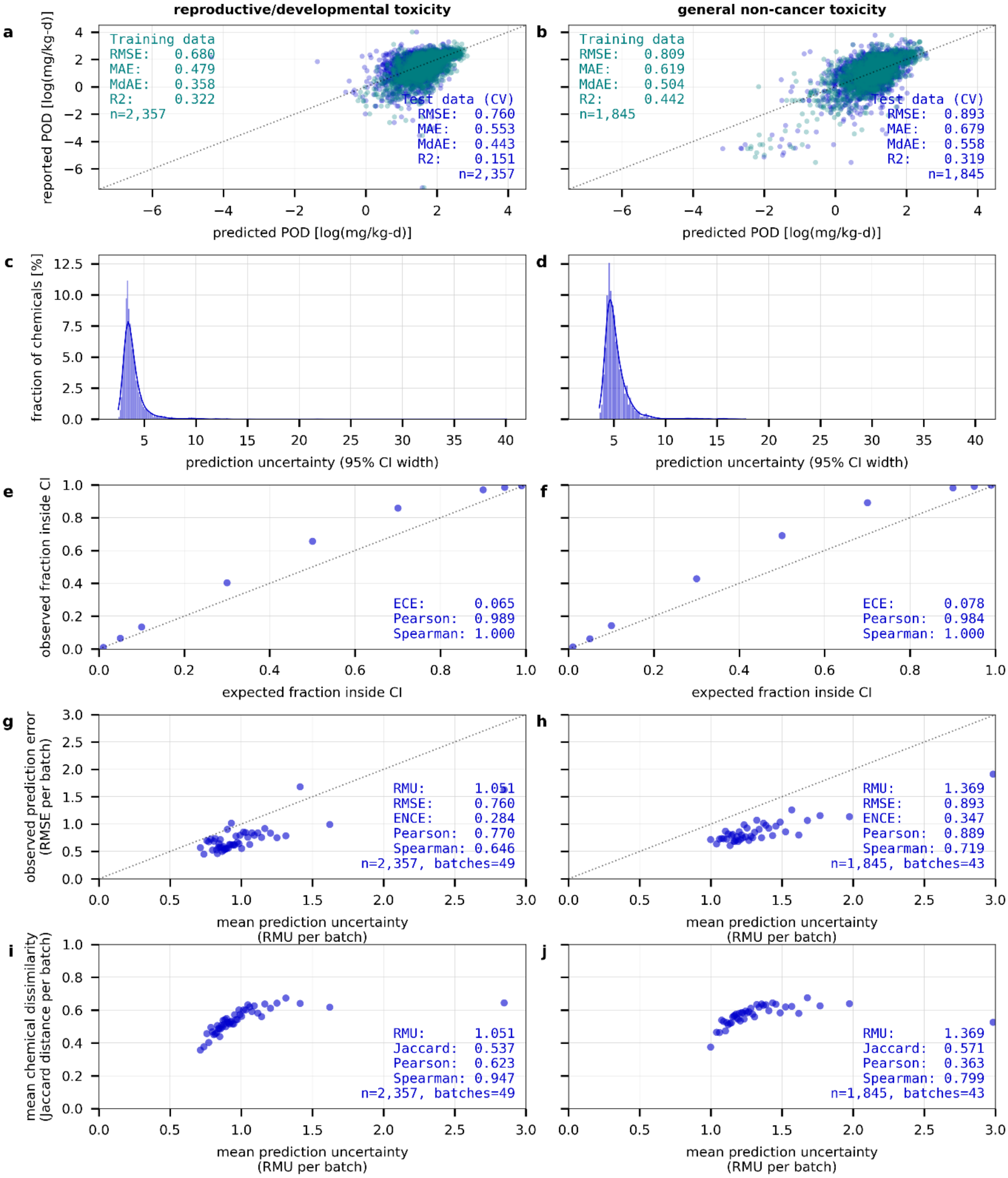

Supplementary Figure 12. Prediction performance and uncertainty calibration of the BNN models trained with Morgan fingerprints. **(a, b)** Prediction performance on training data set (green) and cross-validated test data set (blue). **(c, d)** Distribution of prediction uncertainty (95% CI width) **(e, f)** Confidence-based calibration curve **(g, h)** Error-based calibration curve **(i, j)** Distance-based calibration curve. **Abbreviations:** ECE=expected calibration error, ENCE=expected normalized calibration error, CI=confidence interval, CV = cross-validation, Jaccard=mean Jaccard distances from 5 nearest neighbors, MAE=mean absolute error, MdAE=median absolute error, *n*=number of chemicals, Pearson=Pearson correlation coefficient, POD=point of departure, RMSE=root mean squared error, RMU=root mean uncertainty, R2=coefficient of determination, Spearman=Spearman correlation coefficient. Source data are provided as a Source Data file.

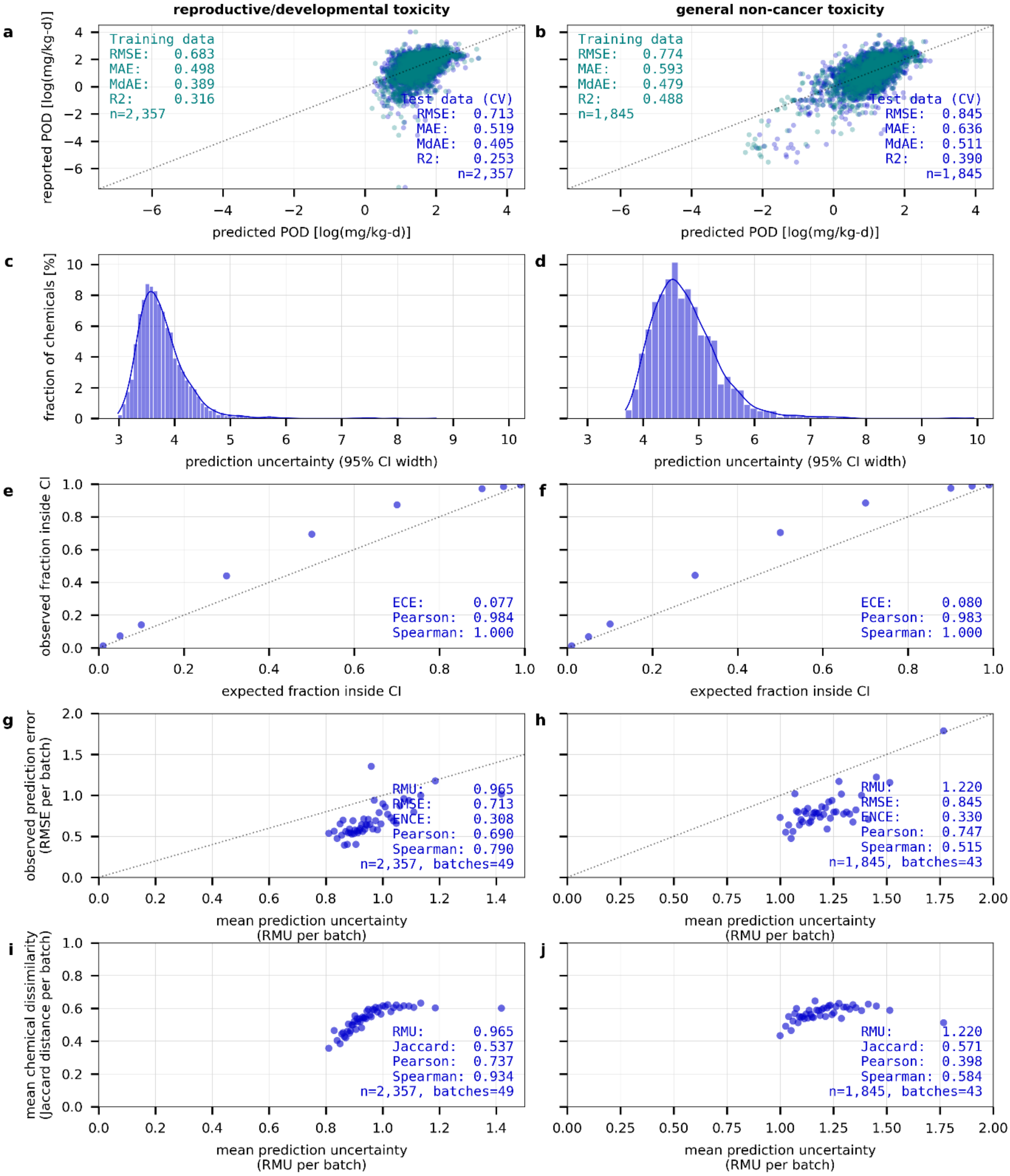

Supplementary Figure 13. Prediction performance and uncertainty calibration of the BNN models trained with CDDD embeddings. **(a, b)** Prediction performance on training data set (green) and cross-validated test data set (blue). **(c, d)** Distribution of prediction uncertainty (95% CI width) **(e, f)** Confidence-based calibration curve **(g, h)** Error-based calibration curve **(i, j)** Distance-based calibration curve. **Abbreviations:** ECE=expected calibration error, ENCE=expected normalized calibration error, CI=confidence interval, CV = cross-validation, Jaccard=mean Jaccard distances from 5 nearest neighbors, MAE=mean absolute error, MdAE=median absolute error, *n*=number of chemicals, Pearson=Pearson correlation coefficient, POD=point of departure, RMSE=root mean squared error, RMU=root mean uncertainty, R2=coefficient of determination, Spearman=Spearman correlation coefficient. Source data are provided as a Source Data file.

Supplementary Figure **14** shows the correlation between prediction medians and uncertainty estimates for reproductive/developmental and general non-cancer PODs across different prediction systems as heatmaps. The underlying scatter plots are shown in Supplementary Figure **15** to Supplementary Figure **18**.

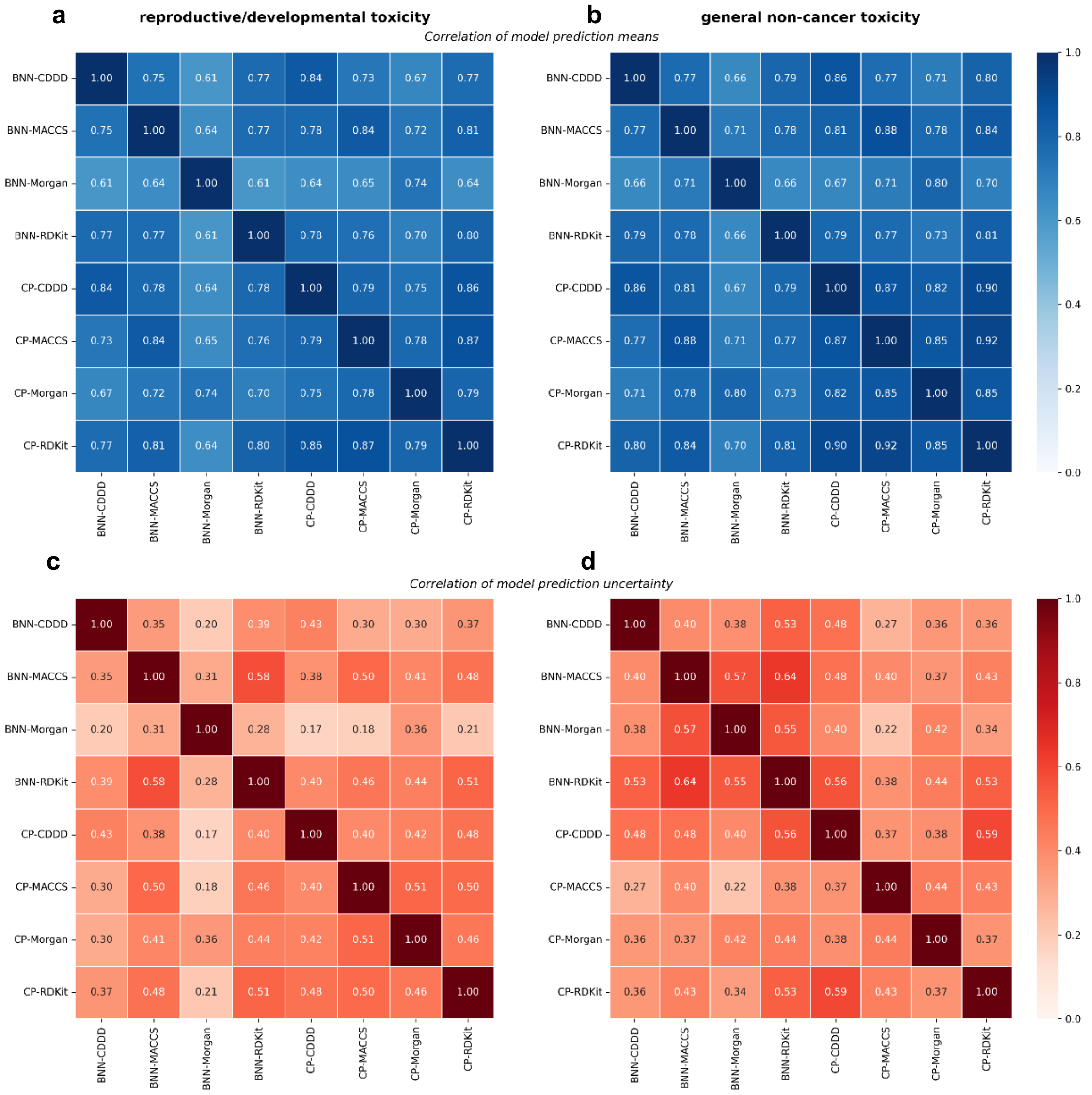

Supplementary Figure 14. Correlation heatmaps of (a, b) median predictions and (c, d) uncertainty estimates (given by 95% CI width) for reproductive/developmental (left) and general non-cancer (right) PODs across different methods and descriptors given by Pearson^2 values. **Abbreviations:** BNN = Bayesian neural network, CDDD = CDDD embedding, CI = Confidence interval, CP = Conformal prediction, MACCS = MACCS keys, Morgan = Morgan fingerprints, RDKit = RDKit descriptors. Source data are provided as a Source Data file.

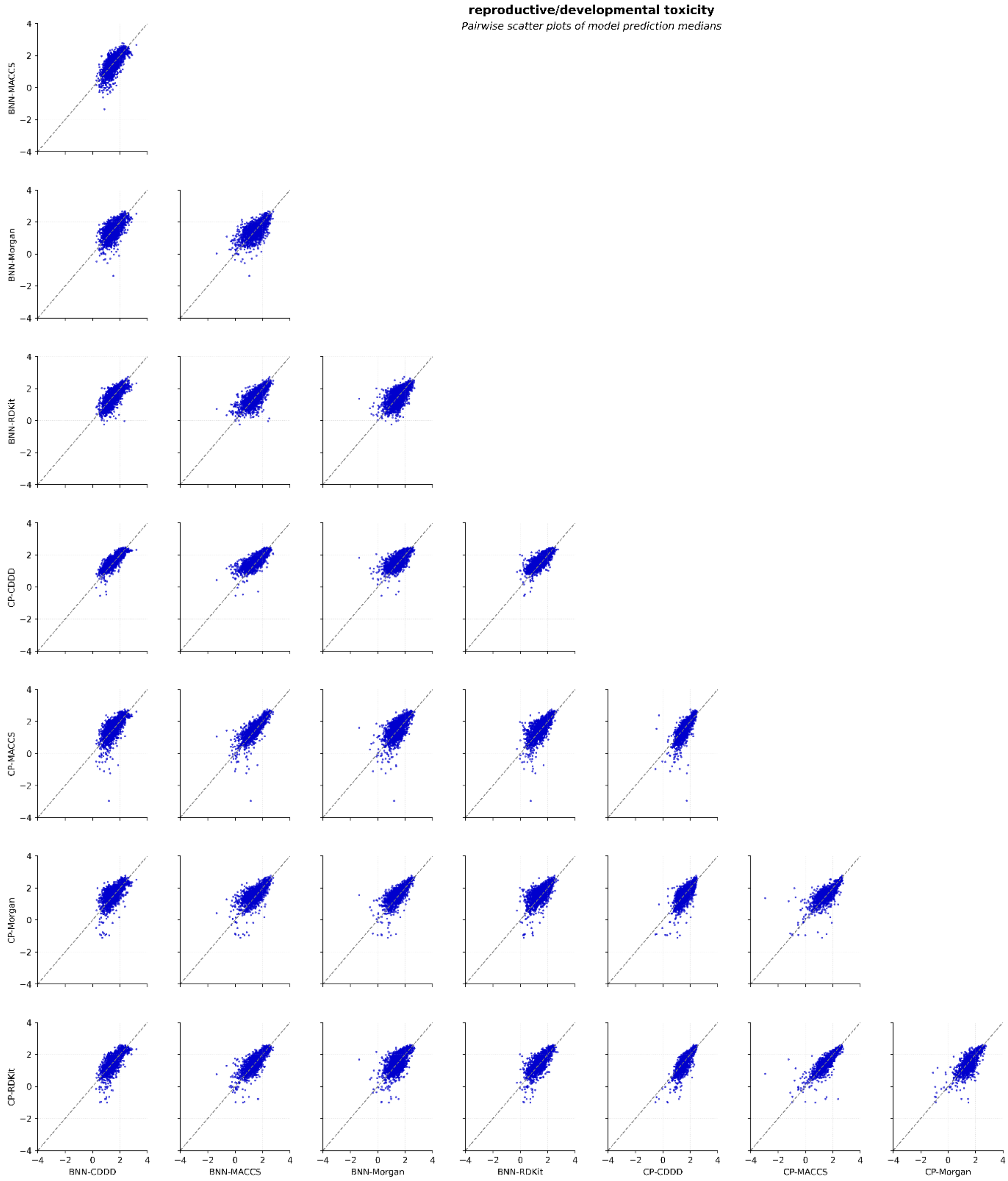

Supplementary Figure 15. Correlation scatter plots of median predictions for reproductive/developmental PODs across different methods and descriptors. **Abbreviations:** BNN = Bayesian neural network, CDDD = CDDD embedding, CP = Conformal prediction, MACCS = MACCS keys, Morgan = Morgan fingerprints, RDKit = RDKit descriptors. Source data are provided as a Source Data file.

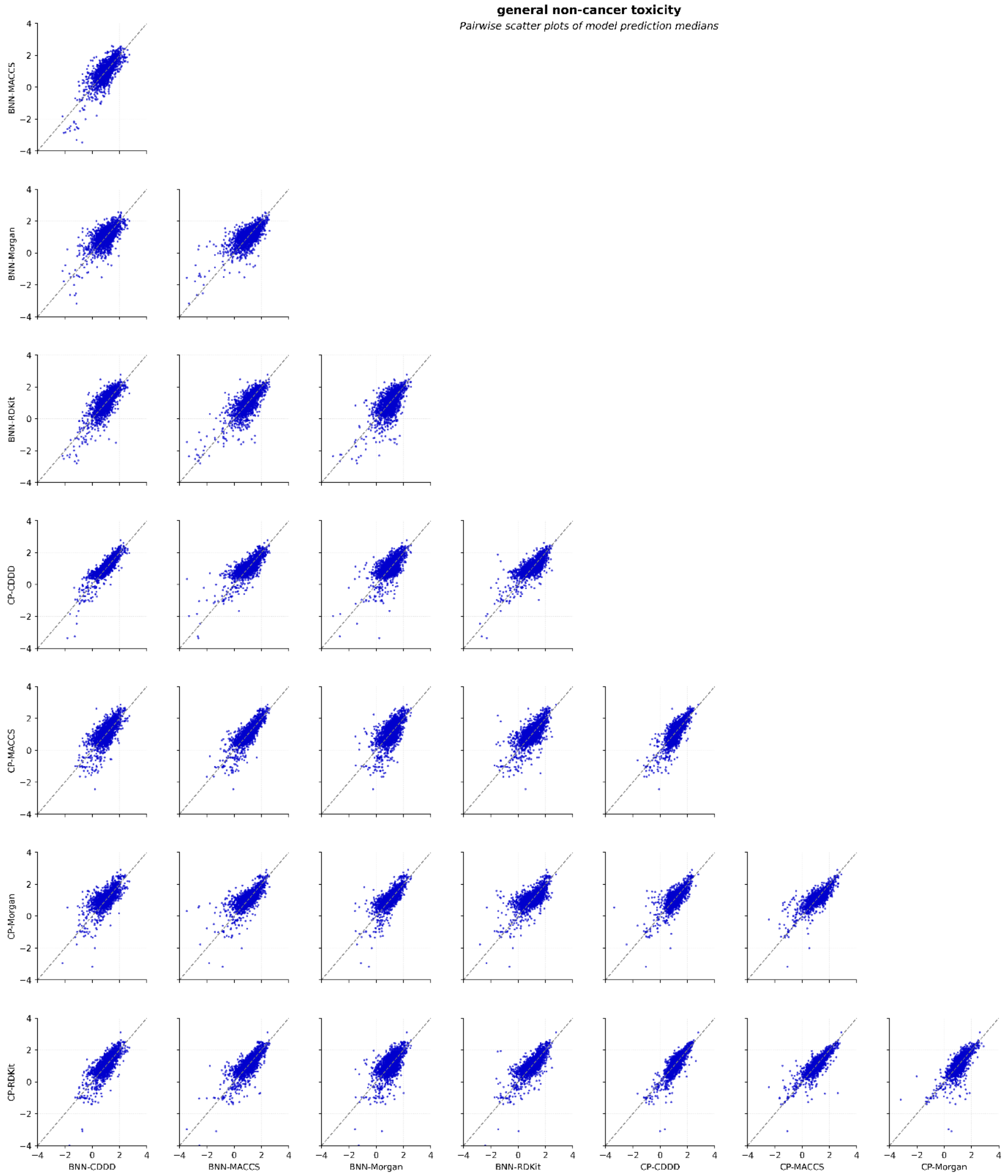

Supplementary Figure 16. Correlation scatter plots of median predictions for general non-cancer PODs across different methods and descriptors. **Abbreviations:** BNN = Bayesian neural network, CDDD = CDDD embedding, CP = Conformal prediction, MACCS = MACCS keys, Morgan = Morgan fingerprints, RDKit = RDKit descriptors. Source data are provided as a Source Data file.

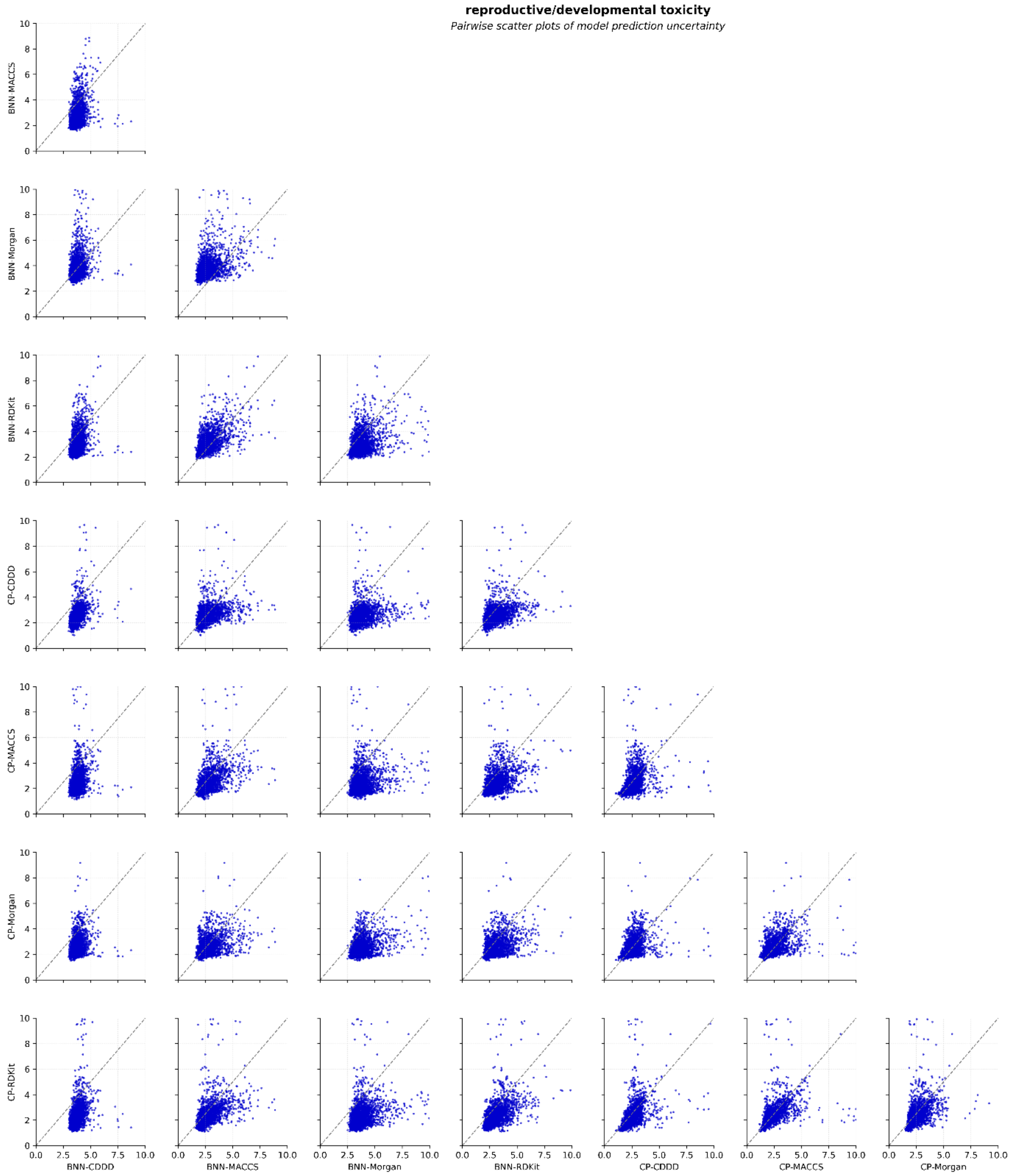

Supplementary Figure 17. Correlation scatter plots of prediction uncertainty (given by 95% CI width) for reproductive/developmental PODs across different methods and descriptors. **Abbreviations:** BNN = Bayesian neural network, CDDD = CDDD embedding, CI = confidence interval, CP = Conformal prediction, MACCS = MACCS keys, Morgan = Morgan fingerprints, RDKit = RDKit descriptors. Source data are provided as a Source Data file.

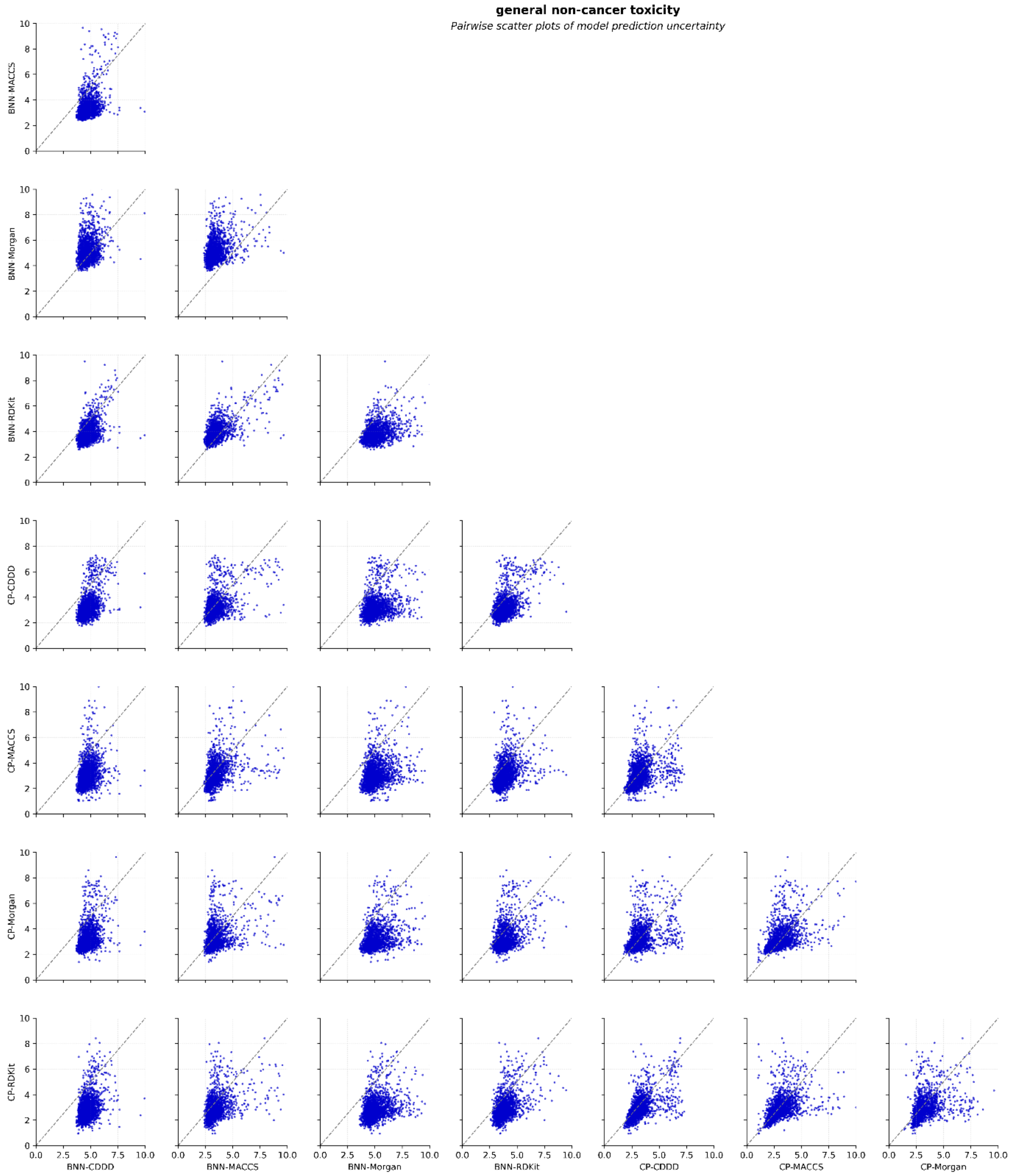

Supplementary Figure 18. Correlation scatter plots of prediction uncertainty (given by 95% CI width) for general non-cancer PODs across different methods and descriptors. **Abbreviations:** BNN = Bayesian neural network, CDDD = CDDD embedding, CI = confidence interval, CP = Conformal prediction, MACCS = MACCS keys, Morgan = Morgan fingerprints, RDKit = RDKit descriptors. Source data are provided as a Source Data file.

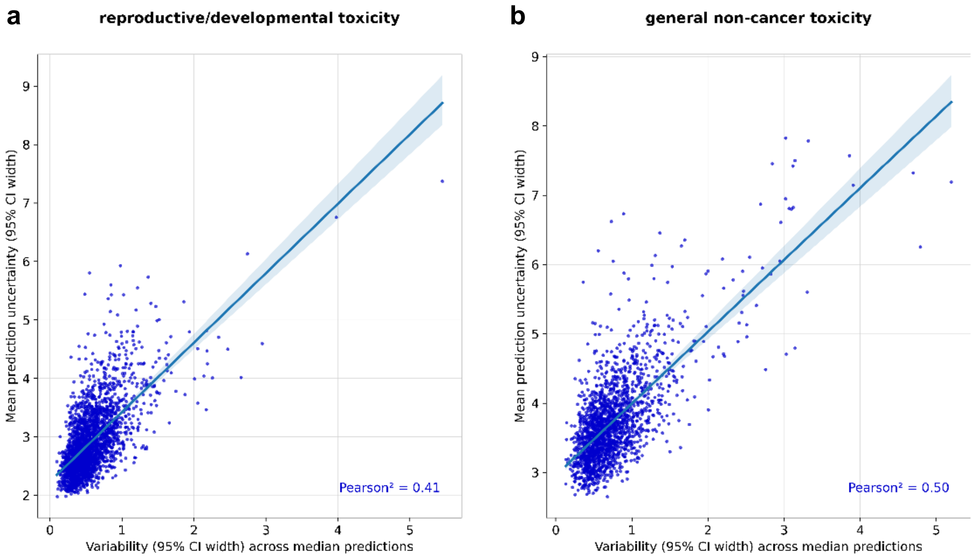

Supplementary Figure 19. Correlation scatter plots with $Pearson^2$ for variability (given as 95% CI width) across median predictions by different models and the prediction uncertainty (given by 95% CI width) averaged across different models predicting (a) reproductive developmental and (b) general non-cancer PODs. Source data are provided as a Source Data file.

Supplementary Figure **20** shows the prediction performance and uncertainty calibration for the models trained with the expanded dataset for predicting POD_{rd} for the non-standardized chemical subset. In comparison, Supplementary Figure **21** and **22** show the prediction performance and uncertainty calibration for the models trained with the standardized dataset when predicting the non-standardized chemicals as a challenging external test set.

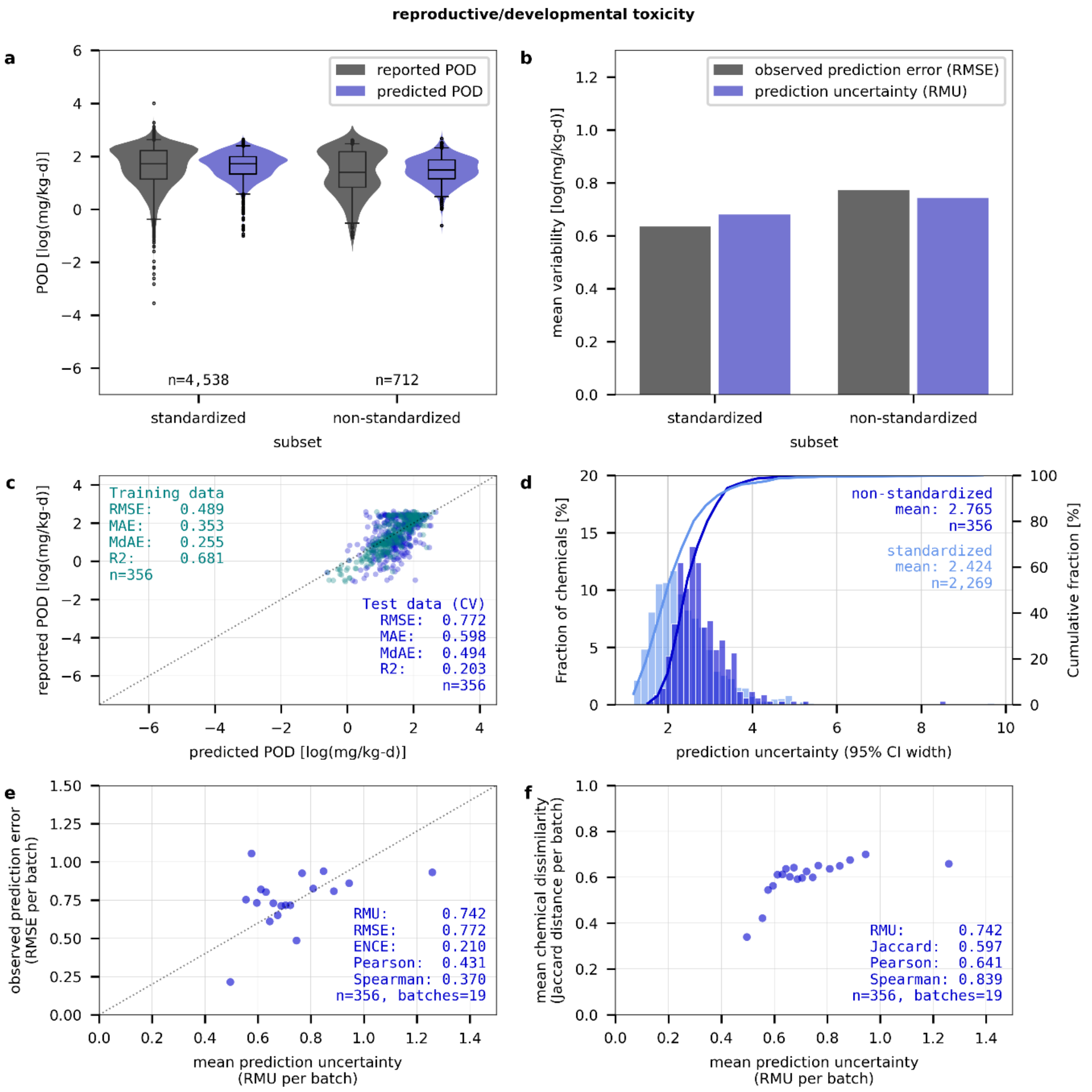

Supplementary Figure 20. Prediction performance and uncertainty calibration for a non-standardized subset of chemicals predicted by CP models trained with an expanded dataset for reproductive/developmental PODs. **(a)** Distribution of reported (grey) and predicted (blue) PODs (based on CV) for standardized and non-standardized chemical subsets. **(b)** The mean prediction uncertainty (RMU, blue) and observed prediction errors (RMSE, grey) for standardized and non-standardized chemical subsets. **(c)** Prediction performance on training data set (green) and cross-validated test data set (blue) for non-standardized subset **(d)** Distribution of prediction uncertainty (95% CI width) for standardized (light blue) and non-standardized (blue) subsets **(e)** Confidence-based calibration curve for non-standardized subset **(f)** Error-based calibration curve for non-standardized subset. Boxplots show the median (center) and interquartile range (boxes) with whiskers extending to the 2.5th and 97.5th percentiles. **Abbreviations:** ENCE=expected normalized calibration error, CI=confidence interval, CV = cross-validation, Jaccard=mean Jaccard distances from 5 nearest neighbors, MAE=mean absolute error, MdAE=median absolute error, *n*=number of chemicals, Pearson=Pearson correlation coefficient, POD=point of departure, RMSE=root mean squared error, RMU=root mean uncertainty, R2=coefficient of determination, Spearman=Spearman correlation coefficient. Source data are provided as a Source Data file.

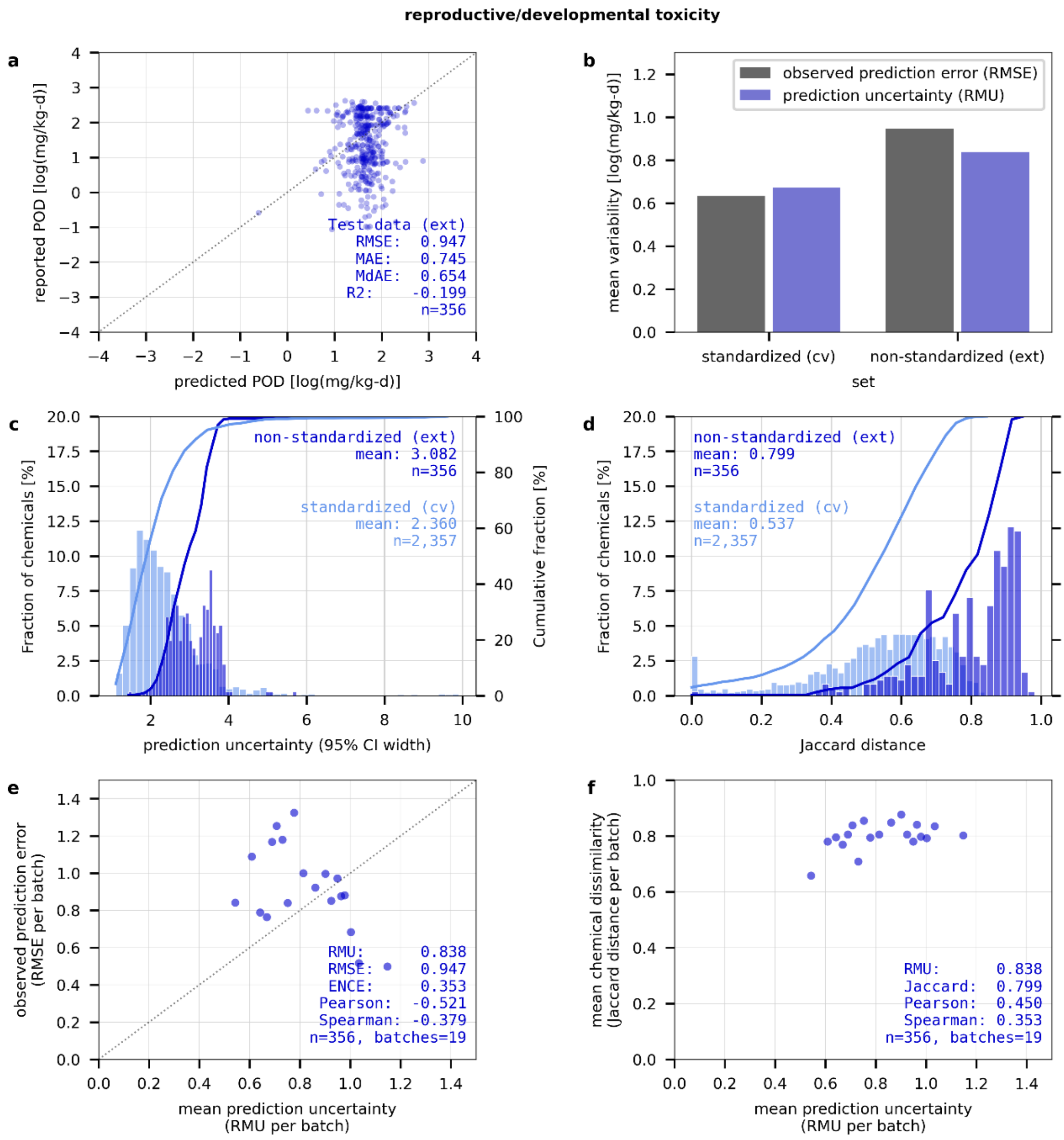

Supplementary Figure 21. External prediction performance and uncertainty calibration for the non-standardized test set predicted by CP models trained with the standardized dataset for reproductive/developmental PODs. **(a)** Prediction performance on the challenging, external test data set of non-standardized chemicals **(b)** The mean prediction uncertainty (RMU, blue) and observed prediction errors (RMSE, grey) for non-standardized chemicals (external test set) compared to standardized chemicals (based on CV). **(c)** Distribution of prediction uncertainty (95% CI width) for non-standardized chemicals (blue) compared to standardized chemicals (light blue, based on CV) **(e)** Confidence-based calibration curve for non-standardized chemicals **(f)** Error-based calibration curve for non-standardized chemicals. **Abbreviations:** ENCE=expected normalized calibration error, CI=confidence interval, CV=cross-validation, Jaccard=mean Jaccard distances from 5 nearest neighbors, MAE=mean absolute error, MdAE=median absolute error, n=number of chemicals, Pearson=Pearson correlation coefficient, POD=point of departure, RMSE=root mean squared error, RMU=root mean uncertainty, R2=coefficient of determination, Spearman=Spearman correlation coefficient. Source data are provided as a Source Data file.

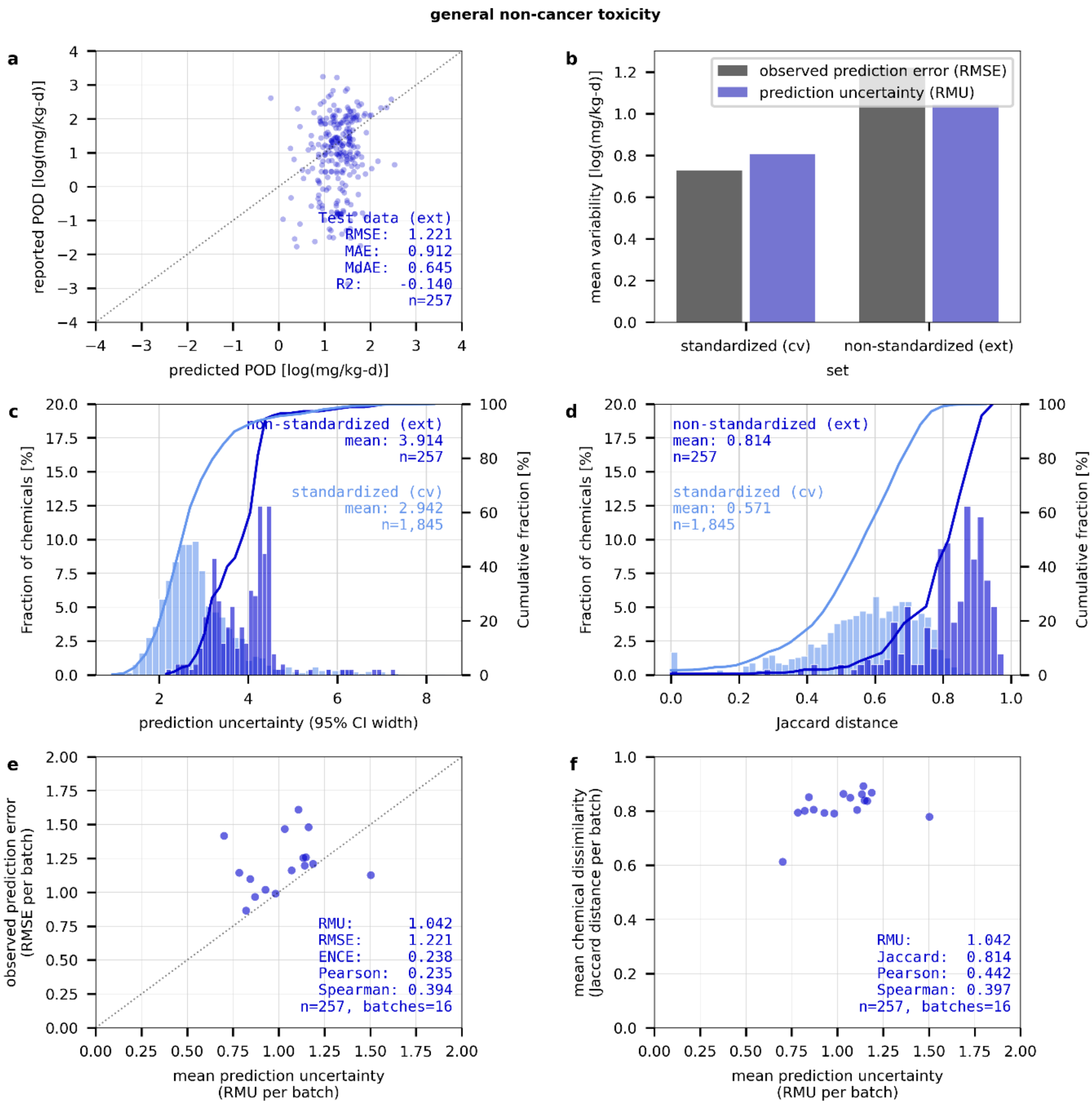

Supplementary Figure 22. External prediction performance and uncertainty calibration for the non-standardized test set predicted by CP models trained with the standardized dataset for general non-cancer PODs. **(a)** Prediction performance on the challenging, external test data set of non-standardized chemicals **(b)** The mean prediction uncertainty (RMU, blue) and observed prediction errors (RMSE, grey) for non-standardized chemicals (external test set) compared to standardized chemicals (based on CV). **(c)** Distribution of prediction uncertainty (95% CI width) for non-standardized chemicals (blue) compared to standardized chemicals (light blue, based on CV) **(e)** Confidence-based calibration curve for non-standardized chemicals **(f)** Error-based calibration curve for non-standardized chemicals. **Abbreviations:** ENCE=expected normalized calibration error, CI=confidence interval, CV=cross-validation, Jaccard=mean Jaccard distances from 5 nearest neighbors, MAE=mean absolute error, MdAE=median absolute error, n=number of chemicals, Pearson=Pearson correlation coefficient, POD=point of departure, RMSE=root mean squared error, RMU=root mean uncertainty, R2=coefficient of determination, Spearman=Spearman correlation coefficient. Source data are provided as a Source Data file.

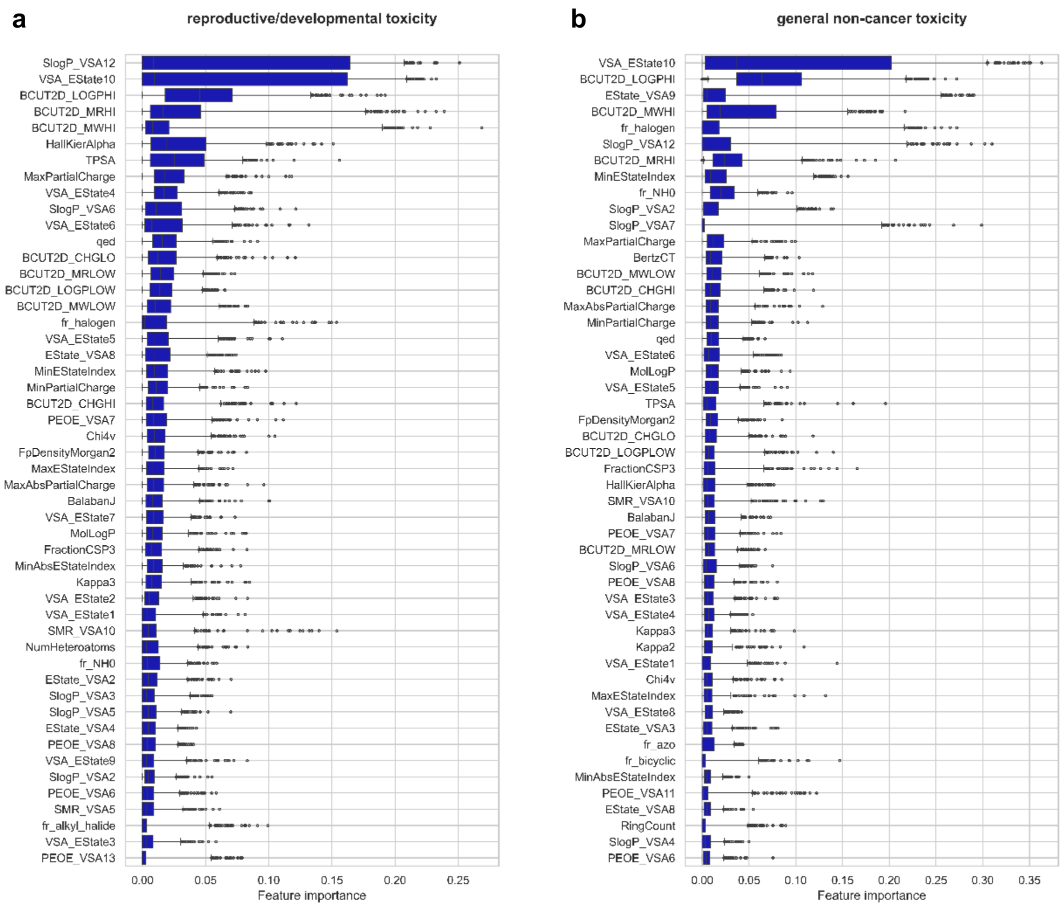

Supplementary Figure 23. Feature importance distributions from 1,000 quantile regression forests underlying the CP models for (a) reproductive developmental and (b) general non-cancer PODs trained with the standardized datasets, shown for the 50 most important features ranked by mean importance. Source data are provided as a Source Data file.

4. Model application

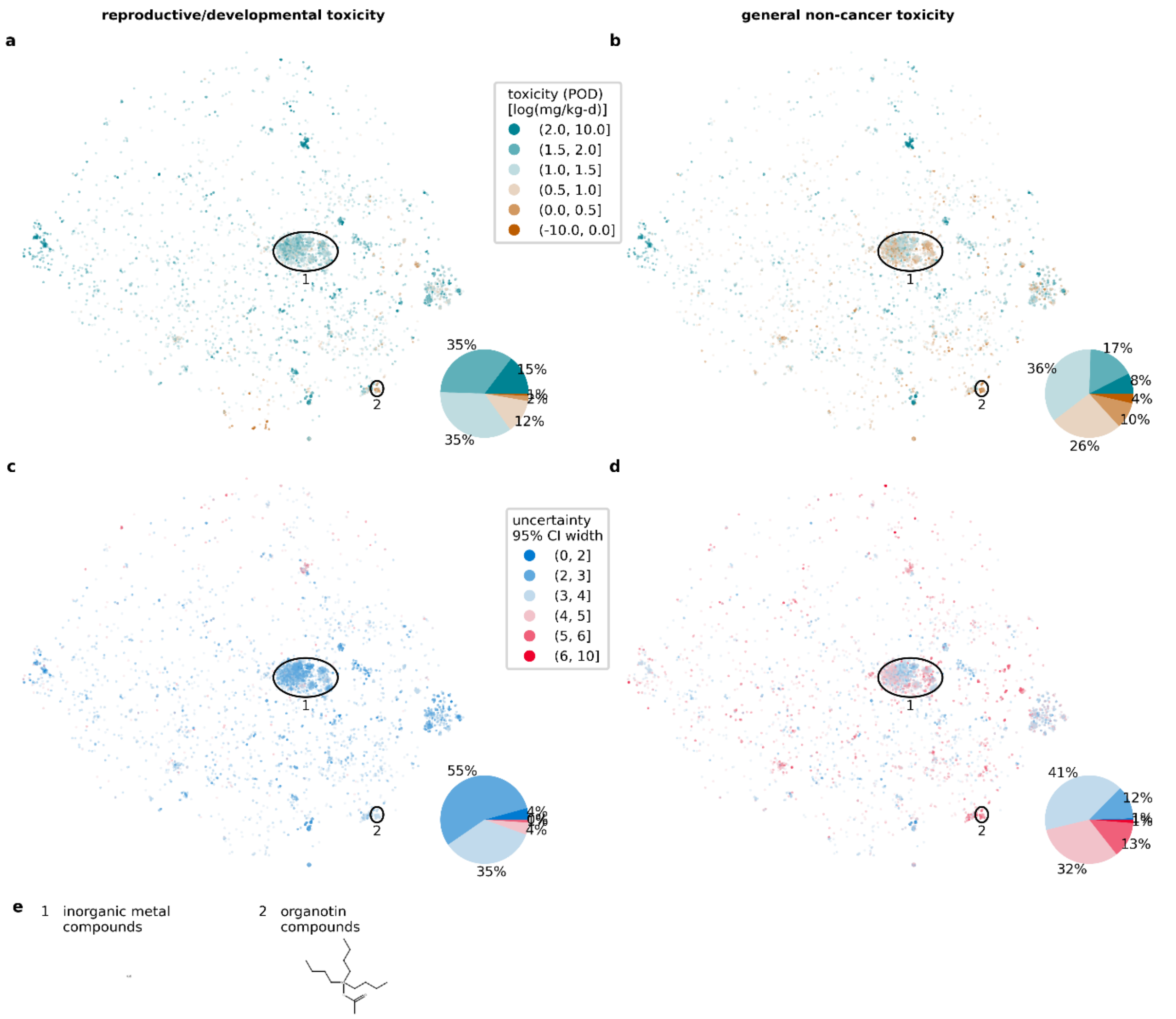

Supplementary Figure 24. 8,054 non-standardized marketed chemicals colored by (a) reproductive/developmental and (b) general non-cancer toxicity given by the predicted $\log_{10}$-scale POD (green to orange color scale) and (c, d) the associated prediction uncertainty given by the $\log_{10}$-scale width of the 95% CI (blue to red color scale) visualized in a two-dimensional chemical space map of 134,114 marketed chemicals with (e) illustrative annotations of high toxicity and high uncertainty hotspots. Pie charts illustrate the percentage of marketed chemicals falling into the respective toxicity and uncertainty levels. **Abbreviations:** CI=confidence interval, POD=point of departure. Source data are provided as a Source Data file.

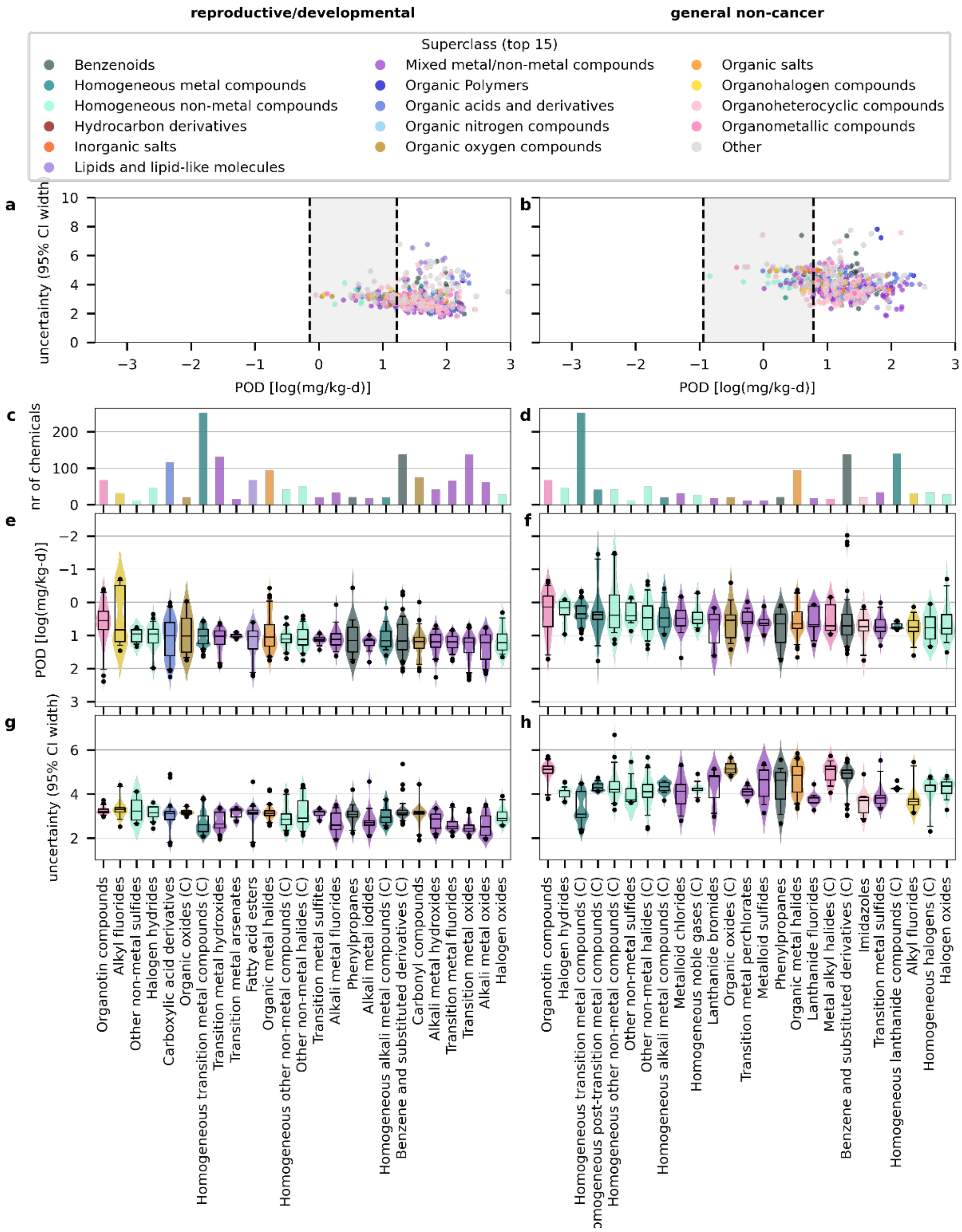

Supplementary Figure 25. Ranking of non-standardized marketed chemicals grouped by chemical classes based on the lowest classification level (subclass, class (C) or superclass (S)) available in the ClassyFire chemical taxonomy showing **(a, b)** the scatterplot of predicted POD and associated uncertainty given by the 95% CI for all available chemical classes and further highlighting the top 25 chemical classes – ranked by their toxicity potential given by the median predicted POD – covering at least 10 marketed chemicals with **(c, d)** the number of chemicals in the top 25 chemical classes, **(e, f)** the distribution of predicted PODs in the top 25 chemical classes and **(g, h)** the distribution of associated prediction uncertainty given by the 95% CI in the top 25 chemical classes. All data points are colored by their respective superclass among the 15 most frequent ClassyFire superclasses across the set of marketed chemicals (see legend). Boxplots show the median (center) and interquartile range (boxes) with whiskers extending to the 2.5th and 97.5th percentiles with underlying sample sizes shown in subfigures (c, d). **Abbreviations**: CI = confidence interval, POD = point of departure. Source data are provided as a Source Data file.

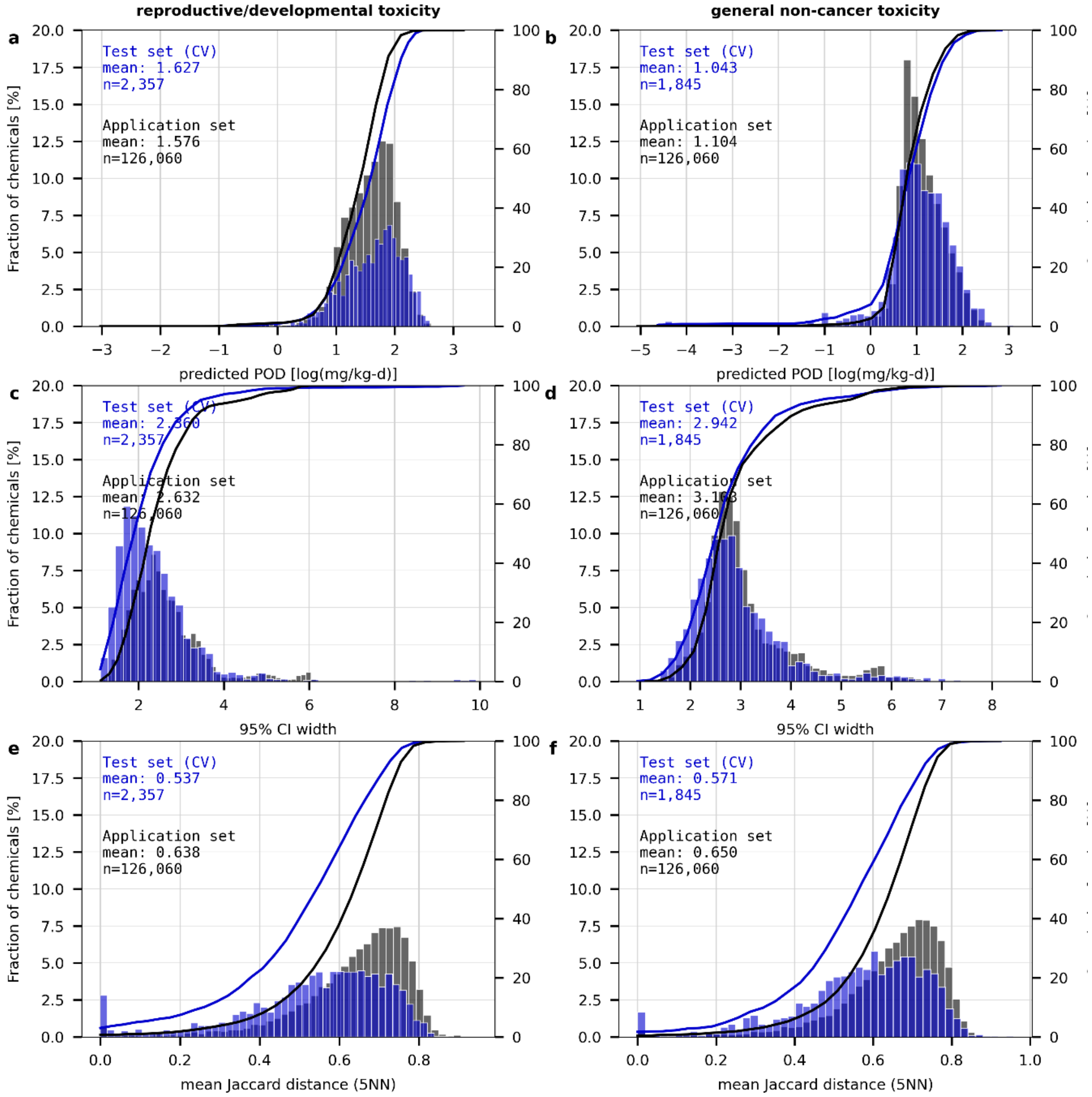

Supplementary Figure 26. Histogram and cumulative distributions of **(a, b)** predicted POD, **(c, d)** prediction uncertainty (95% CI width) and **(e, f)** chemical dissimilarity (mean Jaccard distances) predictions of for for POD_{rd} (left) and POD_{nc} (right) for cross-validated test chemicals and application chemicals (standardized marketed chemicals). Source data are provided as a Source Data file.

References

1. Fantke et al. Exposure and toxicity characterization of chemical emissions and chemicals in products: global recommendations and implementation in USEtox. *International Journal of Life Cycle Assessment* **26,** 899–915 (2021).

2. DeVito et al. The 2022 world health organization reevaluation of human and mammalian toxic equivalency factors for polychlorinated dioxins, dibenzofurans and biphenyls. *Regulatory Toxicology and Pharmacology* **146,** (2024).

3. Williams et al. The CompTox Chemistry Dashboard: A community data resource for environmental chemistry. *J Cheminform* **9,** (2017).

4. Kim et al. PubChem 2023 update. *Nucleic Acids Res* **51,** D1373–D1380 (2023).

5. Swain et al. PubChemPy. Available at https://pubchempy.readthedocs.io/en/latest/index.html (2023)

6. PubChem. PubChem Identifier Exchange Service. (2023). Available at https://pubchem.ncbi.nlm.nih.gov/idexchange/idexchange.cgi

7. Cherkasov et al. QSAR modeling: Where have you been? Where are you going to? *J Med Chem* **57,** 4977–5010 (2014).

8. Gasser et al. Machine learning-based prediction of fish acute mortality: implementation, interpretation, and regulatory relevance. *Environmental Science: Advances* **3**, 8, 1124–1138(2024).

9. RDKit. RDKit: Open-source cheminformatics (2022_09_05). Available at https://doi.org/10.5281/zenodo.7671152 (2022)

10. Winter et al. Learning continuous and data-driven molecular descriptors by translating equivalent chemical representations. *Chem Sci* **10,** 1692–1701 (2019).

11. Durant et al. Reoptimization of MDL keys for use in drug discovery. *J Chem Inf Comput Sci* **42,** 1273–1280 (2002).

12. Rogers et al. Extended-connectivity fingerprints. *J Chem Inf Model* **50,** 742–754 (2010).

13. Cover et al. Approximate formulas for the information transmitted by a discrete communication channel. *IEEE Trans Inf Theory* **24,** (1952).

14. Boser et al. A Training Algorithm for Optimal Margin Classifiers. *Proceedings of the fifth annual workshop on Computational learning theory* 144-152, Association for Computing Machinery (1992)

15. Breiman. Random Forests. *Mach Learn* **45,** 5–32 (2001).

16. Chen et al. *XGBoost: A Scalable Tree Boosting System*. Available at https://github.com/dmlc/xgboost

17. Rosenblatt. The Perceptron: A Probabilistic Model For Information Storage And Organization In The Brain. *Psychol Rev* **65,** (1958).

18. Hüllermeier et al. Aleatoric and epistemic uncertainty in machine learning: an introduction to concepts and methods. *Mach Learn* **110,** 457–506 (2021).

19. Romano et al. Conformalized Quantile Regression. (2019). Preprint at http://arxiv.org/abs/1905.03222

20. Rossellini et al. Integrating Uncertainty Awareness into Conformalized Quantile Regression. Preprint at http://arxiv.org/abs/2306.08693 (2023).

21. Tran et al. Bayesian Layers: A Module for Neural Network Uncertainty. Preprint at http://arxiv.org/abs/1812.03973 (2018).

22. Blundell et al. Weight Uncertainty in Neural Networks. Preprint at http://arxiv.org/abs/1505.05424 (2015).

23. Yang et al. Explainable uncertainty quantifications for deep learning-based molecular property prediction. *J Cheminform* **15,** (2023).

24. Pernot. Properties of the ENCE and other MAD-based calibration metrics. Preprint at http://arxiv.org/abs/2305.11905 (2023).

25. Levi et al. Evaluating and Calibrating Uncertainty Prediction in Regression Tasks. *Sensors* **22,** (2022).

26. Yin et al. Evaluating uncertainty-based active learning for accelerating the generalization of molecular property prediction. *J Cheminform* **15,** (2023).
