# Supplementary material for: Uncertainty-aware machine learning to predict non-cancer human toxicity for the global chemicals market
Source: Nat Commun. 2026 Jan 7;17:647. doi: 10.1038/s41467-025-67374-4 (PMC12816579; doi:10.1038/s41467-025-67374-4)
Supplement: Supplementary file 2 — Reporting Summary [file 41467_2025_67374_MOESM2_ESM.pdf]

nature portfolio

Corresponding author(s): Kerstin von Borries, Peter Fantke

Last updated by author(s): Oct 23, 2025

Reporting Summary

Nature Portfolio wishes to improve the reproducibility of the work that we publish. This form provides structure for consistency and transparency in reporting. For further information on Nature Portfolio policies, see our Editorial Policies and the Editorial Policy Checklist.

Statistics

For all statistical analyses, confirm that the following items are present in the figure legend, table legend, main text, or Methods section.

n/a	Confirmed	
☐	☒	The exact sample size (*n*) for each experimental group/condition, given as a discrete number and unit of measurement
☒	☐	A statement on whether measurements were taken from distinct samples or whether the same sample was measured repeatedly
☐	☒	The statistical test(s) used AND whether they are one- or two-sided<br>*Only common tests should be described solely by name; describe more complex techniques in the Methods section.*
☒	☐	A description of all covariates tested
☐	☒	A description of any assumptions or corrections, such as tests of normality and adjustment for multiple comparisons
☐	☒	A full description of the statistical parameters including central tendency (e.g. means) or other basic estimates (e.g. regression coefficient) AND variation (e.g. standard deviation) or associated estimates of uncertainty (e.g. confidence intervals)
☐	☒	For null hypothesis testing, the test statistic (e.g. *F*, *t*, *r*) with confidence intervals, effect sizes, degrees of freedom and *P* value noted<br>*Give P values as exact values whenever suitable.*
☐	☒	For Bayesian analysis, information on the choice of priors and Markov chain Monte Carlo settings
☒	☐	For hierarchical and complex designs, identification of the appropriate level for tests and full reporting of outcomes
☐	☒	Estimates of effect sizes (e.g. Cohen's *d*, Pearson's *r*), indicating how they were calculated

Our web collection on statistics for biologists contains articles on many of the points above.

Software and code

Policy information about availability of computer code

Data collection	No experimental data was collected as part of this study.
Data analysis	All modelling, data analysis and visualization were performed with code developed in Python 3.11 leveraging publicly available python packages. We used RDKit (version 2022.9.5, https://www.rdkit.org/, ref. 85) to calculate RDKit descriptor, MACCS keys and Morgan fingerprints, and the python package CDDD (version 1.0, https://github.com/jrwnter/cddd, ref. 59) to calculate CDDD embeddings. Conventional machine learning algorithms and training support functions were implemented with scikit-learn (https://scikit-learn.org/, version 1.2.2) and TensorFlow (version 2.13.0, https://www.tensorflow.org/). Uncertainty-aware machine learning algorithms were implemented with the python package UACQR (version 2023-06-08, https://github.com/rrross/uacqr, ref. 77) and TensorFlow Probability (version 0.21.0, https://www.tensorflow.org/probability, ref. 86). Numpy (v1.23.5), Pandas(v1.5.3) and Scipy (v1.10.1) were used for data processing and statistical analysis, and Matpotlib (v3.7.0) and Seaborn (0.13.0) for data visualization. All code used in this study is available on GitHub at: https://github.com/kejbo/poduam. The models are also available via a Shiny web app at: https://dtu-quantitative-sustainability-assessment.shinyapps.io/poduam

For manuscripts utilizing custom algorithms or software that are central to the research but not yet described in published literature, software must be made available to editors and reviewers. We strongly encourage code deposition in a community repository (e.g. GitHub). See the Nature Portfolio guidelines for submitting code & software for further information.

Data

Policy information about availability of data

All manuscripts must include a data availability statement. This statement should provide the following information, where applicable:

- Accession codes, unique identifiers, or web links for publicly available datasets
- A description of any restrictions on data availability
- For clinical datasets or third party data, please ensure that the statement adheres to our policy

In this work, we relied on previously published and publicly available datasets. To train and validate out machine learning models we used toxicity data reported by ref. 5 and ref. 52. We collected SMILES representations from the US EPA CompTox Chemicals Dashboard (version 2.1, https://comptox.epa.gov/dashboard/, ref. 53,64) and PubChem (https://pubchem.ncbi.nlm.nih.gov/, ref. 54). Chemical classifications were obtained with the web-based application ClassyFire (http://classyfire.wishartlab.com/, ref. 63). All data used and generated in this study are available in the GitHub repository PODUAM (v1.0.0) [https://github.com/kejbo/PODUAM] under accession code DOI:10.5281/zenodo.17407072 [https://doi.org/10.5281/zenodo.17407072](ref. 65). The predictions for the large set of marketed chemicals generated in this study are also provided as a structured Excel file in the Supplementary Information. Source data are provided with this paper.

Research involving human participants, their data, or biological material

Policy information about studies with human participants or human data. See also policy information about sex, gender (identity/presentation), and sexual orientation and race, ethnicity and racism.

Reporting on sex and gender	n/a
Reporting on race, ethnicity, or other socially relevant groupings	n/a
Population characteristics	n/a
Recruitment	n/a
Ethics oversight	n/a

Note that full information on the approval of the study protocol must also be provided in the manuscript.

Field-specific reporting

Please select the one below that is the best fit for your research. If you are not sure, read the appropriate sections before making your selection.

☐ Life sciences ☐ Behavioural & social sciences ☒ Ecological, evolutionary & environmental sciences

For a reference copy of the document with all sections, see nature.com/documents/nr-reporting-summary-flat.pdf

Ecological, evolutionary & environmental sciences study design

All studies must disclose on these points even when the disclosure is negative.

Study description	This study presents the development and evaluation of uncertainty-aware machine learning models to predict human reproductive/developmental and general non-cancer points of departure (PODs). The models were trained and validated using pre-curated datasets by Aurisano et al. (2023) complemtened by DeVito et al. (2024) which derived human-equivalent points of departure from publicly available in vivo animal experimental data. The total number of units available for model training and evaluation after data pre-processing were n=2,357 for reproductive/developmental toxicity and n=1,845 for general non-cancer toxicity. Different model algorithms were trained in combination with different sets of molecular descriptors and molecular fingerprints, namely RDKit descriptors, MACCS keys, Morgan fingerprints and CDDD embeddings. Model evaluation was based on k-fold cross validation (k=10) providing replicate estimates of prediction performance. Outcome metrics include R^2 and RMSE for predictive accuracy and three types of calibration curves to assess uncertainty estimates, comparing performance along interactions of model algorithms and training features. No in vivo or in vitro experiments were conducted as part of this study.
Research sample	The data sets by Aurisano et al. (2023) contained human-equivalent toxicity points of departure (PODs) via oral exposure for 10,155 chemicals, wherein n=6,703 chemicals with reproductive/developmental PODs and n=7,354 with general non-cancer PODs. These human-equivalent PODs were derived from a subset of a total of 427,506 in vivo test records in the ToxValDB, selecting only records related to mammalian species, oral exposure routes, and three effect levels (NOAEL, LOAEL, BMDL). These data were then extrapolated to probabilistic human-equivalent benchmark doses (using endpoint- and species-specific extrapolations) to generate surrogate regulatory PODs, representing potential toxicity in the general human population. Toxic equivalency factors (TEFs) reported by DeVito et al. (2024) were used to derive general non-cancer PODs for 26 additional dioxin-like chemicals by multiplying the provided TEFs with the POD for 2,3,7,8-Tetrachlorodibenzodioxin from Aurisano et al. (2023).

Sampling strategy	No experimental data was collected as part of this study.<br>For model evaluation, k-fold cross-validation was performed using stratified sampling based on the target variable (PODs).
Data collection	No experimental data was collected as part of this study.
Timing and spatial scale	No experimental data was collected as part of this study.
Data exclusions	The subsets used for model training and evaluation included only chemicals with unique SMILES representations and a minimum of four underlying data points to reduce data-related uncertainty. Model training was further restricted to chemicals that could be standardized according to the protocol by Mansouri et al. (2018), while non-standardized chemicals were used within an extended training set and as challenging external test sets.
Reproducibility	All data preprocessing, model development, and evaluation procedures were conducted using open-source tools and are fully reproducible. The source code, along with instructions and scripts to replicate all analyses, has been made available with the manuscript. Model hyperparameters, crossvalidation settings, and random seeds were fixed where applicable to ensure reproducibility.
Randomization	Randomization was applied during data splitting (k-fold cross-validation, calibration set) and model initialization using fixed random seeds to ensure reproducibility. Stratified sampling based on the target variable (PODs) was employed to maintain distributional balance across folds.
Blinding	n/a

Did the study involve field work? ☐ Yes ☒ No

Reporting for specific materials, systems and methods

We require information from authors about some types of materials, experimental systems and methods used in many studies. Here, indicate whether each material, system or method listed is relevant to your study. If you are not sure if a list item applies to your research, read the appropriate section before selecting a response.

Materials & experimental systems

n/a	Involved in the study	
☒	☐	Antibodies
☒	☐	Eukaryotic cell lines
☒	☐	Palaeontology and archaeology
☒	☐	Animals and other organisms
☒	☐	Clinical data
☒	☐	Dual use research of concern
☒	☐	Plants

Methods

n/a	Involved in the study	
☒	☐	ChIP-seq
☒	☐	Flow cytometry
☒	☐	MRI-based neuroimaging

Plants

Seed stocks	n/a
Novel plant genotypes	n/a
Authentication	n/a
